# Advances in chimeric antigen receptor-natural killer cell therapy: from mechanisms and preclinical studies to clinical application

**DOI:** 10.3389/fonc.2025.1759796

**Published:** 2026-01-19

**Authors:** Tianyuan Ren, Fengjiang Wang, Xuan Liu, Jun Guo, Sitan Xie

**Affiliations:** 1 ShanDong YinFeng Academy of Life Science, Jinan, Shandong, China; 2 YinFeng Biological Group., LTD, Jinan, Shandong, China

**Keywords:** CAR-NK, chimeric antigen receptor (CAR), hematological malignancies, natural killer cells, solid tumor

## Abstract

Chimeric antigen receptor T-cell therapy (CAR-T) has revolutionized cancer treatment, yet its application remains limited by high costs, safety concerns, and challenges in solid tumors. Natural killer (NK) cells offer a promising alternative due to their innate tumor-killing capacity, diverse cell sources, lower risk of graft-versus-host disease and cytokine release syndrome, and potential for “off-the-shelf” production. This review synthesizes recent advances in CAR-NK, focusing on NK-specific CAR engineering strategies, preclinical models across hematological and solid malignancies, and the latest clinical trials up to 2025. We highlight distinctive CAR-NK optimization approaches, such as integration of Fc-binding domains, cytokine armoring, and strategies to overcome tumor microenvironment mediated resistance, that distinguish CAR-NK from CAR-T platforms. Key challenges, including insufficient *in vitro* expansion, manufacturing scalability barriers, *in vivo* persistence, and the immunosuppressive effects of the tumor microenvironments (TME), as well as their corresponding potential technical solutions, are critically analyzed. By integrating the latest translational insights, this review aims to provide a forward-looking perspective on CAR-NK as a next-generation immunotherapeutic modality.

## 1 Introduction

### 1.1 Current status and limitations of CAR-T

With the growing number of chimeric antigen receptor T-cell (CAR-T) products entering the market, adoptive immune cell therapy has garnered substantial attention. As of September 2025, 14 CAR-T products have secured global marketing approval (**Table 1**) (1–10), 7 of which are approved for use in China, a milestone that solidifies the country’s pivotal role in the global CAR-T landscape.

**Table 1:** Approved CAR-T products.

Name	Target	Manufacturer	Disease	Country	References
Kymriah	CD19	Novartis	B-ALL/B-NHL	USA	(1)
Yescarta	CD19	Kite Pharma	B-NHL	USA	(1)
Tecartus	CD19	Kite Pharma	R/R MCL/B-ALL	USA	(2)
Breyanzi	CD19	Bristol Myers Squibb	LBCL	USA	(1)
Abecma	BCMA	Bristol Myers Squibb	R/R MM	USA	(3)
Carteyva	CD19	JW Therapeutics	R/R LBCL	China	(4)
Carvykti	BCMA	LEGEND Biotech	R/R MM	USA	(5)
Fucaso	BCMA	IASO Biotherapeutics	R/R MM	China	(6)
NexCAR19™	CD19	ImmunoACT	R/R B-cell malignancies/B-ALL	India	(7)
Zevor-cel	BCMA	Carsgen Therapeutics	R/R MM	China	(8)
Yuanduida	CD19	Juventas Cell Therapy Ltd	R/R B-ALL	China	(9)
Aucatzyl	CD19	University College London/Autolus	R/R B-ALL	USA/Europe	(10)
HICARA	CD19	Shanghai Hengrundasheng Biotechnology Co., Ltd.	R/R LBCL	China	(4)

B-ALL, B-cell precursor acute lymphoblastic leukemia; B-NHL, B-cell non-Hodgkin lymphoma; R/R, relapsed/refractory; MCL, Mantle cell lymphoma; MM, Multiple myeloma; LBCL, Large B-cell lymphoma.

Despite these advances, autologous CAR-T is hampered by inherent limitations. These include elaborate, cost-prohibitive manufacturing processes with prolonged turnaround times, and a substantial risk of graft-versus-host disease (GvHD) (11). Additionally, CAR-T is prone to exhaustion (12), which can trigger severe cytokine release syndrome (CRS) (13) and neurotoxicity (14). Their therapeutic efficacy against solid tumors also remains suboptimal, primarily due to the immunosuppressive nature of the tumor microenvironment (TME) and the poor infiltration capacity of CAR-T into tumor tissues. These unmet clinical needs have spurred intensive exploration of alternative adoptive immune cell-based therapeutic strategies.

### 1.2 Mechanisms and advantages of CAR-NK

Since Kuwana (1987) (15) and Gross (1989) (16) first proposed the concept of chimeric antigen receptors (CARs), CAR-based cellular therapies have expanded across multiple disease fields, from hematological and solid tumors to autoimmune disorders (17). The clinical success of CAR-T has further accelerated the development of CAR-natural killer (CAR-NK) cell technology.

NK cells inherently eliminate tumor cells independent of prior antigen priming, thus CAR-NK is widely regarded as a highly effective strategy for tumor immunotherapy (18). While CAR structure design for NK cells shares similarities with CAR-T, both involve genetic element delivery and *ex vivo* expansion of engineered cells (19), and the unique biological characteristics of NK cells endow CAR-NK with distinct advantages over CAR-T.

Multiple sources are exploited for generating allogeneic CAR-NK, including peripheral blood (PB) from healthy donors (20, 21), umbilical cord blood (UCB) (22), induced pluripotent stem cells (iPSCs) (21, 23, 24), and established cell lines (e.g., NK-92) (25, 26). This diverse sourcing supports the development of “off-the-shelf” CAR-NK products, theoretically reducing manufacturing costs and overcoming the limitation of autologous cell unavailability for some cancer patients (27).

CAR-NK also exhibits a more favorable safety profile and robust therapeutic efficacy. Specifically, it carries a lower risk of CRS, immune effector cell-associated neurotoxicity syndrome (ICANS), and GvHD. Notably, CAR-NK can recognize and eliminate tumor cells lacking major histocompatibility complex (MHC) expression (28), a feature that reduces GvHD risk and facilitates allogeneic transplantation. This safety advantage stems from differential cytokine secretion, as CAR-NK primarily secretes interferon (IFN)-γ and granulocyte-macrophage colony-stimulating factor (GM-CSF) (29), whereas CAR-T produces pro-inflammatory cytokines interleukin (IL)-1, IL-6, IL-10, tumor necrosis factor (TNF)-α, monocyte chemoattractant protein-1 (MCP-1) linked to CRS and severe neurotoxicity (30).

**Figure 1:**
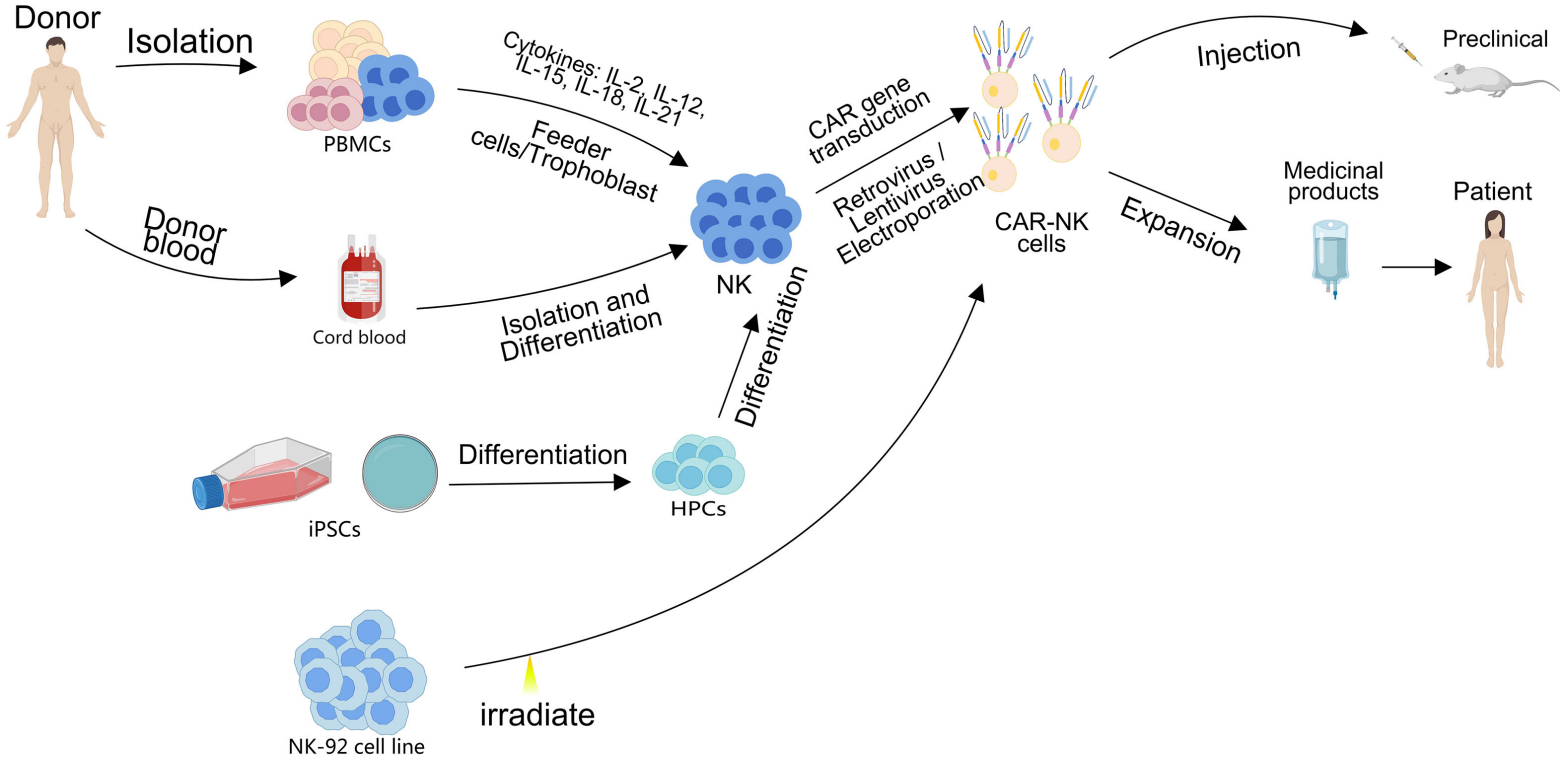
Schematic diagram illustrating the preparation and clinical application of CAR-NK derived from multiple cellular sources, including peripheral blood mononuclear cells (PBMCs), UCB, iPSCs, and the NK-92 cell line.

Endogenously, NK cells distinguish malignant cells via surface receptors [e.g., activating/inhibitory killer cell immunoglobulin-like receptors (KIRs)] (31), enabling “self-non-self” discrimination and avoiding off-tumor cytotoxicity against normal cells (32). CAR-NK eliminates tumor cells through two key mechanisms, intrinsic cytotoxicity via granzyme and perforin release (33, 34), and antibody-dependent cellular cytotoxicity ADCC (35). The workflow of adoptive CAR-NK therapy for cancer is illustrated in **Figure 1**.

Collectively, the accessibility of multiple cellular sources, superior safety profile, and diverse tumor-eliminating mechanisms of CAR-NK overcome the critical barriers of autologous CAR-T and expedite translational progress from preclinical evaluation to clinical application.

### 1.3 Scope and novelty of this review

This review focuses on CAR-NK, a rapidly evolving field with distinct advantages over CAR-T. Its novel scope encompasses four core aspects.

First, Synthesis of research advances through 2025, including preclinical breakthroughs and ongoing clinical trials for hematological malignancies and solid tumors.

Second, highlights of NK-specific CAR engineering innovations, such as Fc-binding domain modification (CD16 optimization) (36–39), cytokine armoring (membrane-bound IL-15 expression) (40–42), NK-specific co-stimulatory domain incorporation (DNAM1, 2B4) (43–45), and TME-resistant engineering (TGF-β receptor knockout, CXCR1/CXCR4 overexpression) (46–50) to overcome solid tumor barriers.

Third, systematic summary of preclinical and clinical progress in major solid tumors (18, 39, 51–53), e.g., hepatocellular carcinoma, ovarian cancer, gastric cancer, brain tumors, prostate cancer, triple-negative breast cancer, as well as innovative combination therapies, CAR-NK combined with oncolytic viruses (54) or photosensitizing agents, and 3D tumor model validations (17, 55).

Fourth, proposal of actionable solutions to key technical bottlenecks includes autocrine IL-15 engineering NKX101, FT576 and suicide switch design for *in vivo* persistence, metabolic reprogramming, chemokine receptor modification and dual-target CAR construction for TME suppression, and feeder-cell-free expansion and non-viral transduction CRISPR/Cas9 combined with transposons for industrialization (56, 57).

This review synthesizes cutting-edge research and bridges the translational gap between basic science and clinical practice, serving as a comprehensive reference for researchers and clinicians in the field.

## 2 NK cell biology and sources

NK cells, a critical component of the innate immune system, play a pivotal role in tumor immunosurveillance and antiviral defense. Their unique biological traits and diverse cellular sources form the foundation for advancing CAR-NK therapy.

### 2.1 Biological characteristics of NK cells

NK cells are large granular lymphocytes derived from bone marrow hematopoietic stem cells (HSCs). Their maturation follows a sequential differentiation pathway, as HSCs differentiate into NK precursor cells, which further develop into immature NK cells and ultimately into functional mature NK cells (58). This process is regulated by a cytokine cascade including IL-15, IL-7, and stem cell factor (SCF) (19, 58).

In terms of tissue distribution, NK cells primarily reside in PB, accounting for 5%–20% of total lymphocytes (59). They also populate various lymphoid and non-lymphoid tissues (e.g., lung, liver, spleen, bone marrow, lymph nodes) and exhibit tissue-specific functional properties (60, 61). Human NK cells are divided into two major subsets based on CD56 expression levels, namely CD56^bright^ and CD56^dim^. The CD56^bright^ subset, representing an early maturation stage, localizes mainly in secondary lymphoid organs and exerts immunomodulatory effects via secreting cytokines such as IFN-γ, tumor TNF, and GM-CSF (62–64). In contrast, the CD56^dim^ subset predominates in PB and spleen, characterized by potent cytotoxicity against tumor and infected cells. This subset highly expresses CD16 (FcγRIIIA), the receptor mediating ADCC (62–64).

NK cell cytotoxicity is tightly regulated by a balance between activating and inhibitory receptors. Activating receptors (e.g., NKG2D, NKp30, NKp44, NKp46) recognize stress-induced ligands on tumor or infected cells to trigger cytotoxicity. Inhibitory receptors (e.g., KIRs, NKG2A, CD94) bind to major MHC class I molecules on normal cells to prevent off-tumor cytotoxicity (31, 32). This dual-receptor system enables NK cells to distinguish self from non-self without prior antigen sensitization, a key feature differentiating them from adaptive immune cells such as T cells (65, 66).

### 2.2 Sources of NK cells for CAR-NK

A core advantage of CAR-NK is the availability of multiple cellular sources, which facilitates the development of off-the-shelf therapeutic products.

#### 2.2.1 PB-derived NK cells

PB from healthy donors is a widely used NK cell source. PB-derived NK cells (PB-NK) can be isolated via density gradient centrifugation followed by depletion of T cells, B cells, and monocytes (21, 22). They exhibit robust cytotoxicity and functional stability, making them suitable for autologous or allogeneic transplantation. However, PB-NK cells are limited by donor-dependent expansion variability and the requirement for large blood volumes to achieve clinical-scale production (19, 31).

#### 2.2.2 UCB-derived NK cells

UCB is a rich source of immature NK cells with high proliferative potential and low immunogenicity. UCB-derived NK cells (UCB-NK) can be efficiently expanded *ex vivo* using cytokine cocktails or feeder cells and display cytotoxicity comparable to PB-NK cells (23). Additionally, UCB is readily accessible from cord blood banks, eliminating the need for donor recruitment and simplifying manufacturing workflows (19, 23). A key limitation is the low NK cell yield per unit volume of UCB, which necessitates efficient expansion strategies to reach therapeutic doses.

#### 2.2.3 iPSC-derived NK cells

iPSCs provide a renewable, standardized source of NK cells. Stepwise cytokine and growth factor induction enables iPSCs to differentiate into NK cells (iPSC-NK), supporting large-scale production of homogeneous cell populations (22, 24, 25). iPSC-NK cells exhibit consistent phenotypic and functional traits across batches, overcoming the donor variability associated with PB-NK and CB-NK cells. Moreover, genetic modifications (e.g., HLA-E knockout, CD47 blockade) can be introduced at the iPSC stage to enhance tumor targeting and reduce immune rejection (22, 37). A critical challenge is the incomplete maturation of iPSC-NK cells, which may lead to impaired *in vivo* cytotoxicity (19, 67).

#### 2.2.4 NK cell lines

Established NK cell lines (e.g., NK-92, NK-92MI, YT) are extensively used in preclinical and clinical research. NK-92 cells, derived from a non-Hodgkin lymphoma patient, can be indefinitely expanded *in vitro* and exert potent cytotoxicity against a broad spectrum of tumor cells (26, 27). They are amenable to genetic modification and large-scale production, making them ideal candidates for off-the-shelf therapy. However, NK-92 cells lack MHC class I expression, which may increase immune rejection risk, and clinical application therefore typically requires irradiation to abrogate *in vivo* proliferation (26, 31).

## 3 CAR structure and engineering strategies

### 3.1 Basic components and evolution of CARs

CARs are synthetic fusion proteins typically composed of four core elements, an extracellular domain (ECD), a hinge region, a transmembrane domain (TMD), and an intracellular domain (ICD). As the antigen-binding domain (ABD), the ECD mediates MHC-unrestricted recognition of tumor-specific antigens. Positioned extracellularly, the hinge region acts as a flexible linker between the ECD and TMD, regulating the spatial orientation and overall conformation of the CAR molecule to directly impact antigen-binding efficacy.

The TMD anchors the receptor in the plasma membrane, with sequences often derived from CD3ζ, CD4, CD8, or CD28, components also found in the ICD. Emerging evidence indicates that TMD composition influences CAR expression, molecular stability, immune synapse formation, and even endogenous signal dimerization (67). The ICD is critical for downstream signaling, usually containing both a co-stimulatory domain (from the CD28 family, e.g., CD28, ICOS, or the TNF receptor family, e.g., 4-1BB, OX40, CD27) and a primary signaling domain. These two components synergize to generate robust intracellular activation signals (67).

### 3.2 Research progress in CAR structure design

**Figure 2:**
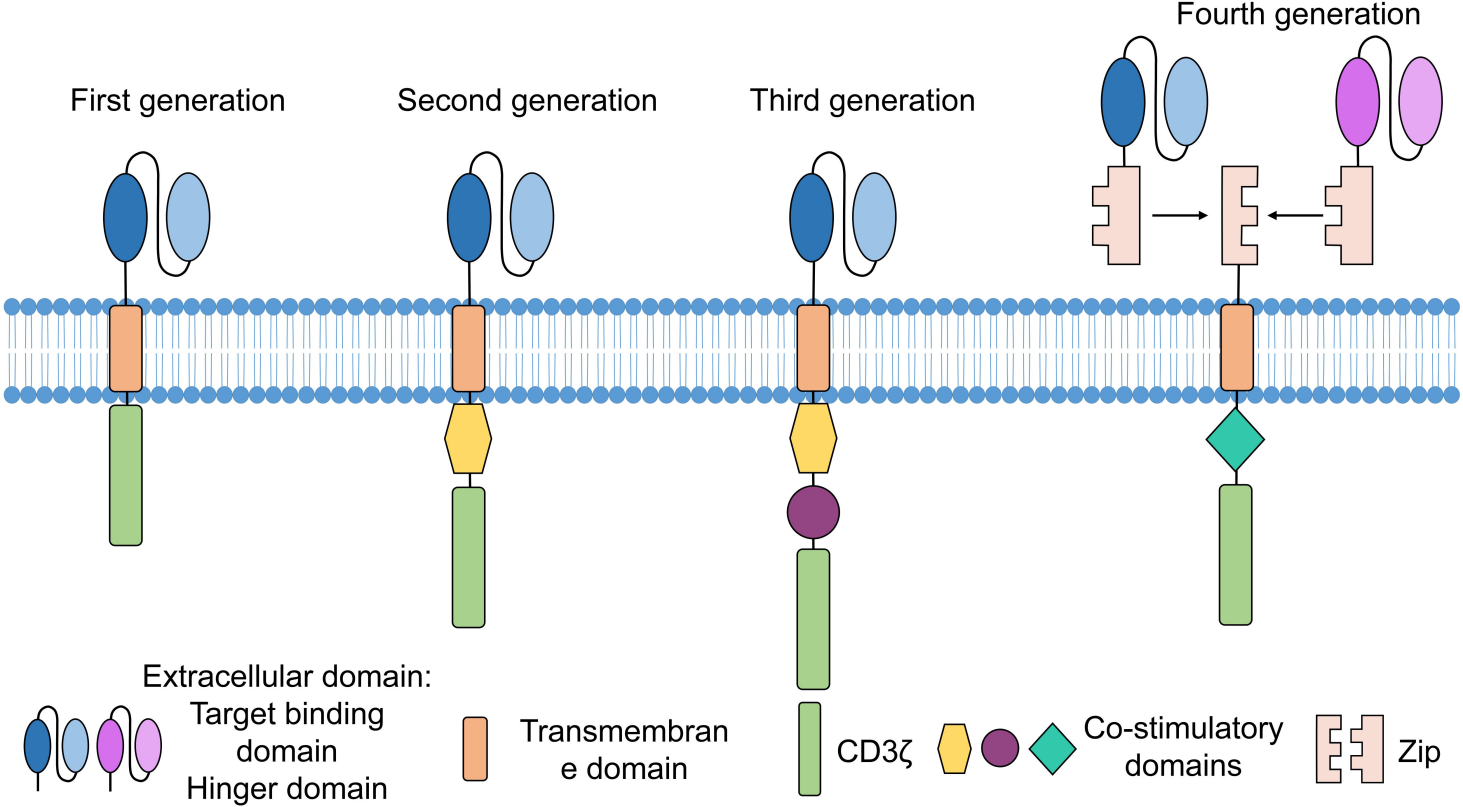
Evolution of CAR generations. First-generation CARs comprise an extracellular antigen-binding domain, a transmembrane domain, and a CD3ζ signaling domain but lack co-stimulatory signals, leading to limited persistence and efficacy. Second-generation CARs integrate a single co-stimulatory domain to provide secondary activation signals, enhancing T cell activation, proliferation, and cytotoxicity. Third-generation CARs incorporate two co-stimulatory domains that act synergistically to further improve T cell persistence and memory formation capacity. Fourth-generation CARs feature a novel “Zip” functional module enabling cytokine secretion or immune cell recruitment, thereby achieving multifunctional expansion and precise therapeutic regulation.

Advancements in CAR engineering have spurred the development of multiple generations of CAR-based immunotherapies, with CAR-T serving as a representative model. Structurally, CAR molecules have evolved through at least four generations (**Figure 2**).

First-generation CARs contain an extracellular antigen-binding domain, a transmembrane domain, and a CD3ζ signaling domain but lack co-stimulatory elements, which renders engineered T cells highly prone to exhaustion. Second-generation CARs integrate a single co-stimulatory domain (e.g., 4-1BB/CD137), enhancing T cell persistence. Notably, most currently approved CAR-T products adopt the second-generation structure (68–70). Third-generation CARs incorporate two co-stimulatory domains (e.g., CD28/4-1BB or CD28/OX40) to prolong cell survival and function, and they are currently under investigation primarily in solid tumor models. Despite these improvements, key challenges remain, including solid tumor targeting, specific antigen recognition, and resistance within the TME. Fourth-generation CARs feature structural innovations such as logic gates, adaptor-dependent circuitry, pharmacological switches, and cytokine secretion modules. Logic gate CARs leverage signal integration from multiple activation or inhibition antigens to improve target recognition and address tumor heterogeneity, for example, dual-target CD19/CD22 CAR-T cells can eliminate cancer cells expressing either antigen (71). Adaptor-dependent CARs rely on supplemental ligands to recognize one or more tumor-associated antigens (TAAs). A bispecific anti-fluorescein isothiocyanate (FITC) adaptor, for instance, bridges CAR-T and tumor cells to induce tumor destruction (72). Additionally, switchable CARs (e.g., those containing the FKBP12 F36V component) allow external regulation of activity via drug administration (73). Cytokine secretion modules are particularly prevalent in CAR-NK, with interleukin-15 (IL-15) integration being a common strategy to enhance NK cell persistence *in vivo*.

### 3.3 Optimization of CAR structures for CAR-NK

**Figure 3:**
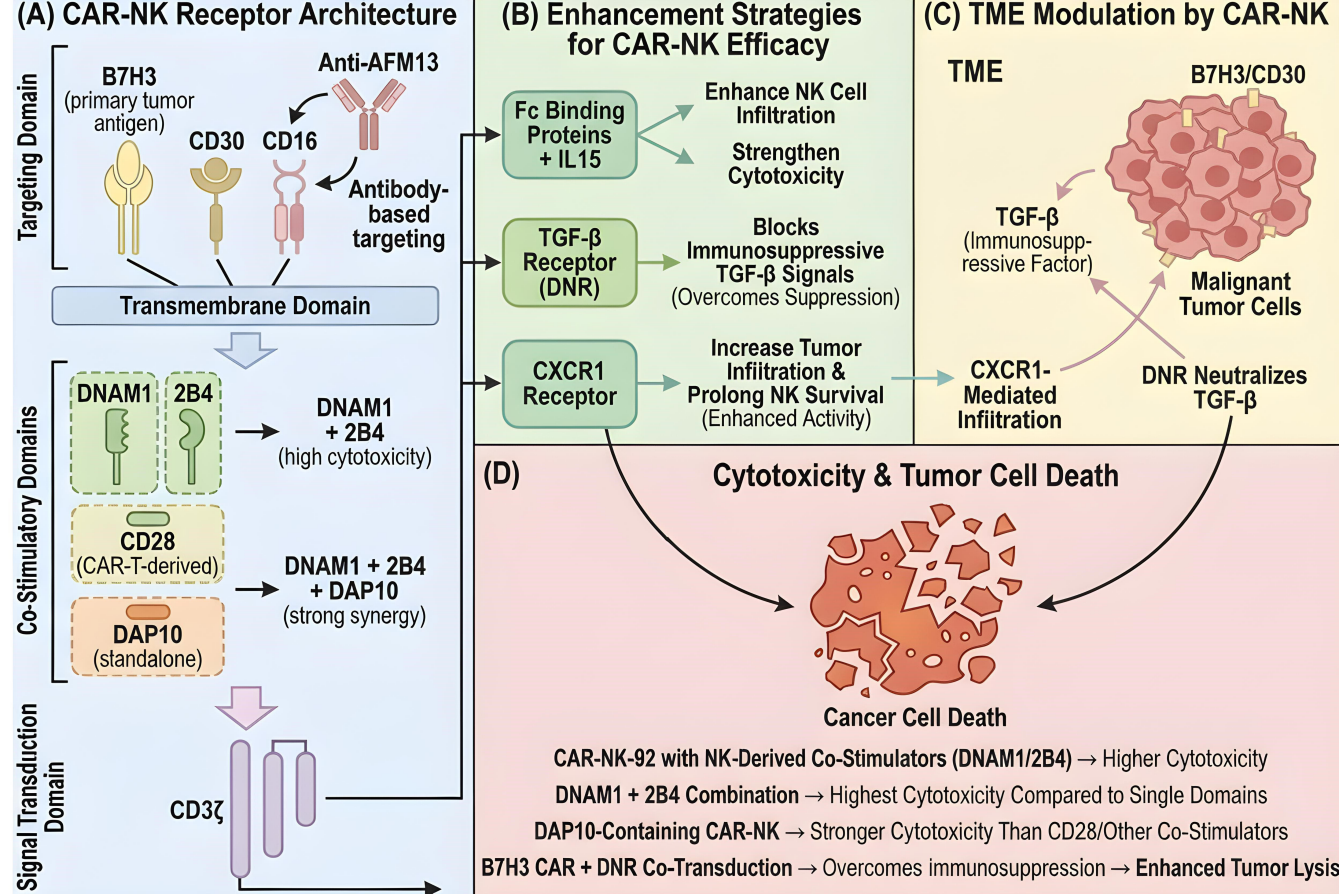
Schematic illustration of CAR-NK receptor architecture, efficacy-enhancing strategies, TME modulation, and induced tumor cell cytotoxicity. **(A)** CAR-NK receptor architecture: The CAR consists of four modules (1): Targeting Domain (2): Transmembrane Domain (3): Co-Stimulatory Domains (4): Signal Transduction Domain. **(B)** Enhancement strategies for CAR-NK efficacy. (1): Fc binding proteins + IL15 (2): TGF-β receptor dominant-negative receptor (DNR) (3): CXCR1 receptor. **(C)** TME modulation by CAR-NK. In the TGF-β-rich immunosuppressive TME, CAR-NK mediates TME remodeling via CXCR1-dependent infiltration and DNR-mediated TGF-β neutralization, while targeting B7H3/CD30 on malignant tumor cells. **(D)** Cytotoxicity and tumor cell death.

Current CAR designs for CAR-NK are largely derived from canonical CAR-T constructs, as both share structural and functional similarities in activation domains (e.g., inclusion of CD3ζ and co-stimulatory regions). However, fundamental differences exist between NK cell and T/B cell activation pathways. Emerging research suggests that customizing CARs with NK cell-specific intracellular domains may further enhance anti-tumor activity and overall therapeutic efficacy (43) (**Figure 3**).

#### 3.3.1 Fc-binding proteins and cytokines

CD16 (FcγRIIIA), a low-affinity IgG receptor expressed on the NK cell surface, plays a pivotal role in mediating ADCC. Incorporating Fc receptor elements into CAR designs has thus become a major research focus. For example, expressing AFM13, a bispecific antibody targeting CD30 and CD16, in UCB-NK cell lines enhances lysis of CD30-positive tumors (36). Dan Kaufman’s group demonstrated that CD16 integration improves the functional performance of iPSC-NK cells (37), while Meng et al. developed a hnCD16-2B4-DAP10-CD3ζ CAR to drive robust ADCC activation (38). Fate Therapeutics has similarly optimized iPSC-NK cell activity using a high-affinity CD16a variant, and Zhang et al. engineered CD33/CD16 CAR-NK with transgenic co-expression of anti-CD16 antibodies to augment cytotoxicity against acute myeloid leukemia (AML) (39).

The *in vivo* longevity and functional activity of CAR-NK can also be enhanced by engineering specific cytokine expression. Mouse models of MM and AML have shown that transgenic IL-15 expression in CAR constructs not only strengthens anti-tumor effects but also extends NK cell survival (40, 41). Furthermore, metabolic adaptability loss is linked to post-therapy resistance, and combining IL-15 incorporation into the CAR backbone with multiple infusion regimens has been shown to overcome such resistance mechanisms (42).

#### 3.3.2 Co-stimulatory and signal transduction domains

Early engineered CARs were designed to mimic T cell receptor signaling by coupling extracellular binding domains to the TCRζ signaling chain. CAR-NK has largely adopted the first four generations of CAR-T, which typically include canonical CD3ζ, 4-1BB, and CD28 co-stimulatory domains. However, comparative studies of five CAR architectures (CD3ζ, CD28-CD3ζ, DNAM1-CD3ζ, 2B4-CD3ζ, DNAM1-2B4-CD3ζ, with the latter two containing NK cell-specific co-stimulatory signals) revealed that CAR-NK-92 cells engineered with NK cell-specific domains (DNAM1 and 2B4) exhibit significantly higher anti-tumor cytotoxicity than those using T cell-derived co-stimulatory domains (43). Among these designs, dual DNAM1/2B4 CAR-NK-92 cells demonstrated the most potent activity (44). Additionally, CAR designs incorporating the DAP10 co-stimulatory motif have also shown enhanced cytotoxic properties (45).

#### 3.3.3 Tumor infiltration and inhibition of TME

A critical challenge for adoptive cell therapy, especially in solid tumors, is achieving efficient tumor infiltration and counteracting the immunosuppressive TME. Overexpressing chemokine receptors such as CXCR4 can enhance the tumor-homing capacity of CAR-NK (46). In peritoneal ovarian cancer (OC) mouse models, CXCR1 upregulation significantly improved CAR-NK infiltration into tumor tissues without compromising cytotoxicity, ultimately boosting anti-tumor efficacy (47).

The TME plays a central role in regulating tumor growth, metastasis, and therapeutic response. Soluble immunosuppressive factors secreted in the TME can directly or indirectly impair NK cell maturation, proliferation, and effector functions, either by acting on NK cells themselves or by modulating the activity of accessory cells such as antigen-presenting cells (APCs). In patient-derived xenograft (PDX) models of glioblastoma multiforme (GBM), TGF-β blockade prevented NK cell dysfunction induced by glioma stem cells (GSCs), enabling superior tumor control (48). To overcome TME-mediated suppression, Chaudhry et al. engineered NK cells co-expressing a B7H3 CAR and a dominant-negative TGF-β receptor (DNR) *in vitro* studies showed that these DNR/B7H3-CAR-NK cells effectively targeted refractory central nervous system tumors including GBM (49). Recent work by Ben-Shmuel et al. further identified cancer-associated fibroblasts (CAFs) as key mediators of TME-driven immune evasion, uncovering novel CAF-dependent mechanisms that inhibit NK cell anti-tumor cytotoxicity (50).

## 4 Preclinical research

### 4.1 CAR-NK in hematological malignancies

Hematological malignancies encompass aggressive diseases (e.g., leukemias, MM, malignant lymphomas) characterized by high lethality, complex treatment regimens, and poor prognoses. Given their prevalence and adverse outcomes, CAR-NK for hematological cancers has become a focal point of intensive research and innovation (51). This section synthesizes preclinical data to analyze core optimization strategies and technical advances across four key dimensions, such as cell source platforms, target selection and optimization, IL-15 utilization, and therapeutic outcome trends (**Table 2**).

**Table 2:** Summary of key preclinical studies of CAR-NK in solid tumors.

Malignancy type	Target antigen	CAR design/Engineering strategy (Conceptual advance)	NK cell source	Key preclinical findings
Hematological Malignancies				
MM	BCMA, GPRC5D	Dual-Targeting CAR	Not Specified	Superior cytotoxicity and reduced tumor recurrence compared to single-target CAR-NK cells.
AML	CD33	Checkpoint Knockout (NKG2A via CRISPR/Cas9)	Not Specified	Enhanced *in vitro* and *in vivo* cytotoxicity by overcoming inhibitory signaling.
AML	CD123	Standard CAR	Not Specified	Induced lower acute toxicity with anti-leukemic efficacy comparable to CAR-T cells.
AML/Broad Hematological	NKG2D	Cytokine Armoring (mbIL-15)	Not Specified (Nkarta’s NKX101)	Robust and sustained cytolytic activity independent of exogenous cytokine support.
AML	CD133/CD16	Bifunctional CAR (Engages endogenous ADCC)	Not Specified	~80% cytotoxicity against AML cells, superior to single-target CD33-CAR-NK cells.
Solid Tumors				
HCC	GPC3	TME Resistance (sPD-L1 blockade)	NK-92	Potent cytotoxicity; co-administration of sPD-L1 variant reversed inhibition and enhanced efficacy.
	GPC3	NK-specific co-stimulation (DNAM1 + 2B4)	Not Specified	Further elevated cytotoxicity of GPC3-CAR-NK cells.
	CD147, GPC3	Bispecific CAR (Dual antigen recognition for safety)	Not Specified	​Effective tumor elimination; no toxicity in CD147 transgenic models, suggesting safety for off-the-shelf use.
	c-MET	Target Validation	Primary NK	Superior cytotoxicity against high c-MET-expressing HCC cells.
OC	MSLN	Standard Target Validation	NK-92	Potent and specific anti-tumor activity *in vitro* and *in vivo*.
	MSLN	Cytokine Signaling Enhancement (Neo-2/15 agonist)	Not Specified	Superior proliferation, cytolytic capacity, and *in vivo* persistence.
	GPC3	Off-the-shelf Platform (iPSC-derived)	iPSC-NK/ILC	Significant survival benefit in models with no acute toxicity or tumorigenicity.
	CD24	Optimized scFv (“SW11”) in 3rd gen CAR	NK-92	Potent, specific activity against OC cell lines and primary patient cells.
	CLDN6	Standard Target Validation	NK-92MI	Robust anti-tumor effects against CLDN6^+^ OC cells.
GC	MSLN	Standard CAR	NK-92	Effective tumor lysis and extended survival in PDX models.
	HER2	Combination Therapy (with apatinib)	NK-92	Efficacy in small tumors; apatinib improved infiltration. Highlights limitation in large, established tumors.
	PD1/NKG2D	Novel Receptor Design (DTCR: PD1-DAP10/NKG2D)	NK-92	​Dramatically augmented cytotoxicity and therapeutic enhancement *in vivo*.
	HER2	Enhanced Innate Immunity (High-affinity, cleavage-resistant CD16)	Placental HSC-NK (CYNK-101)	Next-generation off-the-shelf candidate with enhanced proliferative potential.
GBM	Multiple/CD73	TME Modulation (Local secretion of anti-CD73 scFv)	Not Specified	Overcame TME suppression and attenuated antigen escape via local adenosine pathway inhibition.
	GFR/EGFRvIII	Dual-Targeting CAR	Not Specified	​Significant tumor shrinkage and extended survival after intracranial administration.
	EGFRvIII	Improved Trafficking (CXCR4 overexpression)	Not Specified	Enhanced tumor accumulation and survival benefits.
	HER2	Standard Target Validation	Not Specified	Effective cytolysis and robust tumor growth inhibition in models.
	EGFR	Combination Therapy (with oncolytic virus OV-IL15C)	Off-the-shelf CAR-NK	Synergistic effect, increased immune cell infiltration and persistence.
DIPG	GD2	Standard Target Validation	NK-92	Effective killing of high GD2-expressing DIPG cells; target-dependent efficacy.
Prostate Cancer	PSMA	Combination Therapy (with anti-PD-L1)	NK-92	Enhanced antitumor efficacy in castration-resistant models.
TNBC	CD44v6	Standard Target Validation	NK cells	Targeted therapy for triple-negative breast cancer.

MM, Multiple myeloma; AML, Acute myeloid leukemia; HCC, Hepatocellular carcinoma; OC, Ovarian cancer; GC, Gastric cancer; GBM, Glioblastoma; DIPG, Diffuse intrinsic pontine glioma; TNBC, Triple-negative breast cancer.

#### 4.1.1 Cell source platforms

iPSC-NK and UCB-NK cells exhibit high expansion efficiency and stable tumoricidal activity, making them promising candidates for allogeneic “off-the-shelf” therapies. For example, iPSC-NK cells engineered with HLA-E knockout and CD47 blockade show enhanced evasion of host immune rejection while preserving cytotoxicity (74). As a standardized cell line, NK-92 cells enable mass production but traditionally require preclinical irradiation, limiting their utility. A study reduced this requirement via CRISPR/Cas9-mediated CD56 upregulation, expanding their applicability (75). PB-NK cells are easily accessible but display significant interindividual variability in expansion and functional stability. *Ex vivo* priming with IL-12/IL-18 plus feeder cell co-culture reduces this heterogeneity, improving expansion efficiency by 2.3-fold compared to conventional methods (76).

#### 4.1.2 Target selection and optimization strategies

Validated single-target CAR-NK therapies include CD123 and CD33 for acute myeloid leukemia (AML), BCMA for MM, and CD19 for lymphoma. Notably, CD123-CAR-NK exhibits anti-leukemic efficacy comparable to CAR-T in human hematopoietic stem cell-transplanted AML mouse models, with lower acute toxicity (53). Dual-target strategies represent a critical breakthrough for improving efficacy and reducing recurrence. BCMA/GPRC5D dual-target CAR-NK enhances MM cell killing and reduces tumor escape (42, 77). CD33/CD16 bifunctional CAR-NK achieves an 80% killing rate against AML cells, outperforming single-target regimens (39). Additionally, immune checkpoint knockout (e.g., CRISPR/Cas9-mediated NKG2A deletion) relieves tumor-induced immunosuppression, significantly improving *in vitro* tumoricidal activity and *in vivo* anti-tumor function (52, 77).

#### 4.1.3 IL-15 utilization methods

IL-15 is a core cytokine for sustaining CAR-NK persistence and activity, with membrane-bound IL-15 (mbIL-15) outperforming exogenous recombinant IL-15. For example, Nkarta’s NKX101 (NKG2D-CAR-NK) uses mbIL-15 fused to a CD8α hinge domain to enhance surface retention, extending anti-tumor activity to 12 weeks in AML models (56, 78, 79). Co-expression of mbIL-15 and IL-21 in BCMA-CAR-NK promotes memory-like NK cell formation, with 60% of treated mice remaining tumor-free at 6 months (80, 81). IL-15/IL-15Rα sushi domain fusion also reduces systemic cytokine release while maintaining autocrine signaling (82, 83).

#### 4.1.4 Outcome trends

Combined strategies (dual-target design, immune checkpoint knockout, mbIL-15 modification) exhibit synergistic effects. For example, triple-modified (BCMA/GPRC5D + NKG2A knockout + mbIL-15) CAR-NK cells achieve 90% tumor clearance in MM models, compared to 55% with single-modified cells (42, 84). Allogeneic platforms (iPSC-NK, CB-NK, NK-92) are core future directions, as they maintain efficacy while meeting “off-the-shelf” clinical demands. CRISPR-edited iPSC-NK cells (CD38 and CD47 knockout) avoid daratumumab-induced fratricide, enabling combination with standard-of-care drugs (85). Compared to CAR-T, CD19-targeted CAR-NK for B-ALL reduces CRS by 80% (86).

### 4.2 CAR-NK cell therapy in solid tumors

Solid tumor immunotherapy faces critical challenges, including profound TME immunosuppression and a lack of specific TAAs. Robust preclinical research is positioning CAR-NK as a safer, more efficacious alternative, with mechanistic insights driving innovation. This section analyzes advances across cell source platforms, target selection, TME breakthrough strategies, and IL-15 application, alongside outcome trends and innovative directions (**Table 2**).

#### 4.2.1 Cell source platforms

NK-92 cells are the most widely used in solid tumor preclinical studies due to their standardization. For example, GPC3-CAR-NK-92 achieves 65% tumor regression in hepatocellular carcinoma (HCC) PDX models (87). MSLN-CAR-NK-92 enhances OC infiltration via matrix metalloproteinase (MMP) secretion (88, 89). iPSC-NK cells engineered with CXCR4 and CD137 co-stimulation show a 3-fold improvement in blood-brain barrier penetration in GBM models (90). PB-NK cells modified with c-MET-CAR and IL-15 exhibit 70% cytotoxicity against c-MET-high NSCLC cells, though interindividual variability persists (91). Placental HSC-derived CYNK-101 (high-affinity CD16) shows 40% longer persistence in gastric cancer models than UCB-NK (92–94).

#### 4.2.2 Target selection and antigen heterogeneity mitigation

Core targets for solid tumors are increasingly well-defined. CD147/GPC3 dual-target CAR-NK reduces antigen escape by 50% in HCC PDX models (95). CLDN6-CAR-NK exhibits 85% cytotoxicity against CLDN6 positive OC cells with no cross-reactivity to normal tissues (96, 97). B7-H3/EGFRvIII dual-target CAR-NK achieves 70% tumor shrinkage in intracranial GBM models (98). CAR-T-validated targets (e.g., NKG2D ligands, FOLR1) are also being translated to CAR-NK, as FOLR1-CAR-NK shows 60% cytotoxicity in OC, comparable to FOLR1-CAR-T but with lower toxicity (99).

#### 4.2.3 IL-15 application and persistence enhancement

Beyond mbIL-15 modification, IL-2Rβγ agonists (e.g., Neo-2/15) combined with CAR-NK represent a novel direction. For example, Neo-2/15-MSLN-CAR-NK exhibits superior proliferation and cytotoxicity in OC cell lines and patient-derived organoids, with reduced immunosuppressive factor secretion and improved survival in animal models (100). ALT-803 (IL-15/IL-15Rα fusion) combined with MSLN-CAR-NK improves proliferation by 3.5-fold in pancreatic cancer models (101). Oncolytic viruses (e.g., OV-IL15C, which expresses an IL-15/IL-15R fusion protein) also enhance CAR-NK infiltration and persistence in brain tumors via TME remodeling and sustained IL-15 delivery (54).

#### 4.2.4 TME breakthrough strategies and outcome trends

Chemokine receptor engineering is a key strategy to overcome TME barriers, and CXCR4-overexpressing EGFRvIII-CAR-NK increases GBM tumor accumulation by 4-fold (102). CCR2b-modified MSLN-CAR-NK improves migration to pancreatic cancer stroma (103). TME modulation also includes adenosine pathway blockade, as CAR-NK secreting a CD73-inhibiting scFv elevate intratumoral ATP levels by 60%, reversing immunosuppression in HCC (104). Combination therapies are expanding rapidly, including anti-VEGF (bevacizumab) combined with CAR-NK in OC, which reduces vascular density and improves tumor penetration (105). CAR-NK paired with PD-L1 inhibitors, low-dose chemotherapy, or oncolytic viruses to enhance tumoricidal effects. Stroma-targeting strategies (e.g., against cancer-associated fibroblasts) to indirectly improve TME conditions. Therapeutic outcomes show CAR-NK is most effective in early-stage solid tumor models, as HER2-CAR-NK eliminates 95% of small-volume gastric cancer xenografts, and combination with low-dose paclitaxel extends efficacy to large tumors (60% shrinkage) (106). However, efficacy in large-volume tumors is limited by poor infiltration, suggesting CAR-NK is optimal for early intervention or combined debulking therapy. Promising advances also include PSMA-CAR-NK plus PD-L1 inhibitors for prostate cancer and CD44v6-CAR-NK for triple-negative breast cancer. Advanced technologies (photosensitizer-loaded NK cell drug delivery, 3D tumor spheroids, multiphoton imaging) provide new tools to overcome TME suppression and enable real-time evaluation of CAR-NK behavior (107, 108).

### 4.3 Comparison of CAR-NK strategies between hematological malignancies and solid tumors

Preclinical CAR-NK development reveals fundamental strategic differences dictated by the distinct biological contexts of hematological malignancies and solid tumors. These differences stem from their core challenges, namely the efficient eradication of disseminated, accessible tumor cells in hematological malignancies versus overcoming TME physical and immunosuppressive barriers to reach targets in solid tumors.

In hematological malignancies, tumor cells circulate in the blood or reside in the bone marrow, making them directly accessible to intravenously infused CAR-NK. Research thus focuses on maximizing effector cell potency and durability, with key strategies including dual-targeting CARs (e.g., BCMA/GPRC5D in MM) (42, 84) to mitigate antigen escape and immune checkpoint knockout (e.g., NKG2A in AML) to amplify activation. “Cytokine armoring” (e.g., mbIL-15 in NKX101) to ensure long-term persistence (56, 78, 79). The goal is to create a potent, persistent “living drug” for systemic eradication of disseminated disease.

Solid tumors present a “fortress-like” challenge, with malignant cells embedded in a TME that excludes and suppresses immune cells. Research priorities shift to navigation and survival in a hostile niche, with key advances including chemokine receptor engineering (e.g., CXCR4 in GBM) (102) to improve tumor homing. Secretion of neutralizing agents (e.g., CD73 scFv in HCC) (104) to resist immunosuppression. Resistance engineering to soluble inhibitors (e.g., TGF-β in HCC) (49). Bispecific CARs and TME-modifying combinations (e.g., anti-angiogenics, oncolytic viruses) to address antigen heterogeneity and physical barriers.

In summary, hematological malignancy strategies center on enhancing intrinsic CAR-NK capabilities for systemic eradication, while solid tumor strategies focus on engineering CAR-NK to overcome extrinsic TME suppression for localized attack. This distinction in challenges and solutions defines the divergent developmental pathways of CAR-NK across cancer types.

## 5 Clinical progress

Compelling preclinical outcomes of CAR-NK research have accelerated the translation of this platform into clinical evaluation.

### 5.1 Hematological malignancies

CAR-NK has shown substantial promise in hematological malignancies, with clinical trials targeting diverse antigens and utilizing multiple NK cell sources (**Table 3**).

**Table 3:** Clinical trial examples of CAR-NK for hematological malignancies or autoimmune disease (information from ClinincalTrials.gov).

NK source	Target	CAR structure	Cancer	Phase	Status	NCT No.	Study start	Country
Unknown	Unknown	Unknown	Hematopoietic/Lymphoma	III	Completed	NCT00833898	Nov. 2008	USA
Unknown	CD30	Unknown	Lymphomas	I/II	Unknown status	NCT02274584	Mar. 2014	China
NK-92	CD7	scFv-CD28-4-1BB-CD3ζ	T cell malignancies/AML	I/II	Unknown	NCT02742727	Mar. 2016	China
NK-92	CD19	scFv-CD28-4-1BB-CD3ζ	Leukemia and Lymphoma	I/II	Recruiting	NCT02892695	Sep. 2016	China
NK-92	CD33	scFv-CD28-4-1BB-CD3ζ	AML	I/II	Unknown	NCT02944162	Oct. 2016	China
Cord blood	CD19	scFv-CD28-CD3ζ-iCasp9-IL15	B-cell lymphoma	I/II	Completed	NCT03056339	Jun. 2017	USA
Unknown	CD19/CD22	Unknown	Refractory B-cell lymphoma	Early Phase I	Unknown	NCT03824964	Feb. 2019	Unknown
Unknown	CD19	Unknown	Refractory B-cell lymphoma	Early Phase I	Unknown	NCT03690310	Mar. 2019	Unknown
Unknown	CD22	Unknown	Refractory B-cell lymphoma	Early Phase I	Unknown	NCT03692767	Mar. 2019	Unknown
NK-92	BCMA	scFv-CD8αTM-4-1BB-CD3ζ	MM	I/II	Unknown	NCT03940833	May. 2019	China
Unknown	CD7	Unknown	Leukemia and lymphoma	I	Unknown	NCT04004637	Aug. 2019	China
Cord blood	CD19	scFv-CD28-CD3ζ-iCasp9-IL15	B-cell lymphoma	I/II	Withdrawn	NCT03579927	Oct. 2019	USA
PB-NK	NKG2D-L	NKG2D-OX40-CD3ζ-mblL-15	R/R AML/MDS	I	Recruiting	NCT04623944	Sep. 2020	USA
Unknown	CD7	Unknown	Hematological malignancies	I	Completed	NCT04538599	Sep. 2020	China
Unknown	CD33/CLL1	Unknown	AML	Early Phase I	Recruiting	NCT05215015	Nov. 2020	China
Unknown	CD7	Unknown	T-Lymphoblastic leukemia	I	Unknown	NCT04480788	Nov. 2020	China
Unknown	CD19	Unknown	NHL	Early Phase I	Not yet recruiting	NCT04639739	Dec. 2020	China
Unknown	CD19	Unknown	B-cell lymphoma	I	Recruiting	NCT04796688	Mar. 2021	China
Cord blood	CD19	Unknown	Lymphoblastic leukemia/NHL	I	Recruiting	NCT04796675	Apr. 2021	China
PB-NK	CD19	Unknown	NHL	I	Recruiting	NCT04887012	May. 2021	China
PB-NK	CD19	CD19 scFv-CD8aTM-OX40-CD3ζ-T2A-IL15	B-cell malignancies	I	Recruiting	NCT05020678	Aug. 2021	USA
Cord blood	BCMA	Unknown	MM	Early Phase I	Recruiting	NCT05008536	Oct. 2021	China
Cord blood	NKG2D-L	Unknown	AML	NA	Terminated	NCT05247957	Oct. 2021	China
iPSC (FT576)	BCMA	scFv-NKG2D-2B4-CD3ζ-IL15/R-hnCD16	MM	I	Recruiting	NCT05182073	Nov. 2021	USA
Unknown	Unknown	Unknown	B-cell leukemia/B-cell lymphoma	I/II	Unknown	NCT04747093	Nov. 2021	China
Unknown	Unknown	Unknown	B-cell acute leukemia/B-cell lymphoma	I	Recruiting	NCT05379647	Nov. 2021	China
PB-NK	CD19	Unknown	Lymphocytic leukemia/NHL	I	Recruiting	NCT05410041	May. 2022	China
Unknown	CD19	Unknown	ALL	I	Completed	NCT05563545	Jul. 2022	China
Cord blood	CD19	Unknown	NHL	I	Recruiting	NCT05472558	Sep. 2022	China
Unknown	CD123	Unknown	R/R AML	Early Phase I	Recruiting	NCT05574608	Oct. 2022	China
Unknown	CD19	Unknown	Relapsed NHL/B-cell lymphoma/B-cell leukemia/DLBCL	I	Recruiting	NCT05487651	Oct. 2022	USA
Unknown	CD19	Unknown	B-cell lymphoma/B-cell leukemia	I/II	Withdrawn	NCT05570188	Oct. 2022	China
Unknown	Unknown	Unknown	AML	I	Recruiting	NCT05601466	Oct. 2022	China
Cord blood	CAR.70	Unknown	B cell lymphoma/MDS/AML	I/II	Recruiting	NCT05092451	Nov. 2022	USA
Unknown	BCMA	Unknown	MM	Early Phase I	Recruiting	NCT05652530	Nov. 2022	China
Unknown	CD19/CD70	Unknown	NHL	I	Recruiting	NCT05667155	Dec. 2022	China
Unknown	CD19	Unknown	Refractory B-cell hematologic malignancies	I	Recruiting	NCT05645601	Dec. 2022	China
Unknown	CD19	Unknown	B-cell lymphocytic leukemia/lymphoma	I/II	Recruiting	NCT05654038	Dec. 2022	China
Unknown	CD33	Unknown	AML	I	Recruiting	NCT05665075	Dec. 2022	China
PB-NK	CD19, CD79 and CD123	Unknown	Myelo or lymphoproliferative disorders	Not applicable	Recruiting	NCT06727383	Dec. 2022	European
Cord blood	CD19/CD70	Unknown	R/R NHL	I/II	Recruiting	NCT05842707	Jan. 2023	China
Unknown	CD19	Unknown	DLBCL	Early Phase I	Recruiting	NCT05673447	Mar. 2023	China
Unknown	CD19	Unknown	Leukemia/B-cell lymphoma	Early Phase I	Recruiting	NCT05739227	Mar. 2023	China
Unknown	NKG2D	Unknown	AML	NA	Recruiting	NCT05734898	Mar. 2023	China
Unknown	BCMA	Unknown	MM	Early Phase I	Recruiting	NCT06045091	Jul. 2023	China
Unknown	CD33/CLL1	Unknown	AML	I	Not yet recruiting	NCT05987696	Aug. 2023	China
Unknown	CD19	Unknown	SLE	Early Phase I	Recruiting	NCT06010472	Aug. 2023	China
Unknown	CD123	Unknown	BPDCN	I/II	Recruiting	NCT06006403	Aug. 2023	China
Unknown	CLL1	Unknown	AML	I	Recruiting	NCT06027853	Sep. 2023	China
CB-NK	CD5	Unknown	Hematological malignancy	I/II	Recruiting	NCT05110742	Nov. 2023	USA
Unknown	CD19	Unknown	R/R NHL	I	Recruiting	NCT05618925	Nov. 2023	USA
Unknown	CD123	Unknown	R/R AML	Early Phase I	Recruiting	NCT06201247	Dec. 2023	China
Unknown	CD19	Unknown	Resistant B-cell ALL	I/II	Not yet recruiting	NCT06631040	Aug. 2024	China
Unknown	CD19/ BCMA	Unknown	SLE	Early Phase I	Recruiting	NCT06792799	Jan. 2025	China
iPSC	CD19	Unknown	Refractory B cell-mediated autoimmune disease	I	Recruiting	NCT06255028	Feb. 2025	USA
iPSC	CD19	Unknown	B-Cell Malignancies	I	Active, not recruiting	NCT05336409	Jan. 2023	USA

AML, Acute myeloid leukemia; MM, Multiple myeloma; MDS, Myelodysplastic syndrome; NHL, Non-hodgkin lymphoma; ALL, Acute lymphoblastic leukemia; DLBCL, Diffuse large B cell lymphoma; SLE, Systemic lupus erythematosus; BPDCN, Blastic plasmacytoid dendritic cell neoplas; iPSC, Induced pluripotent stem cell.

#### 5.1.1 Core target antigens and efficacy breakthroughs

##### 5.1.1.1 CD19

As a well-established target in B-cell malignancies, CD19-directed CAR-NK have demonstrated robust safety and efficacy. A Phase I/II trial of IL-15-secreting CD19-CAR-NK (CAR19-IL-15 NK) in 37 CD19-positive patients achieved a 48.6% objective response rate (ORR) at day 30 without severe toxicities, validating the synergy of CAR targeting and cytokine enhancement (109, 110). This is further supported by 2023 long-term follow-up data (111), which reported a 3-year progression-free survival (PFS) rate of 32.4% in R/R NHL. Globally, ongoing trials include a Chinese Phase II study (NCT04796688) investigating cord blood-derived CD19-CAR-NK, with 2024 interim data (112) showing a 56.7% ORR and median PFS of 14 months. Additionally, 2021 U.S. Phase I data (113) demonstrated that combining CD19-CAR-NK with lenalidomide achieved a 63% ORR in high-risk chronic lymphocytic leukemia (CLL), highlighting the potential of synergistic regimens. Collectively, these studies underscore global efforts to optimize CD19-CAR-NK dosing, patient selection, and combination strategies.

##### 5.1.1.2 BCMA

For R/R MM, BCMA-targeted CAR-NK products have emerged as promising off-the-shelf options. Fate Therapeutics’ iPSC-derived FT576 (BCMA-CAR-NK) exhibited excellent tolerability and efficacy in 2024 Phase I data (114) involving 18 R/R MM patients, achieving a 72.2% ORR, including 38.9% complete response (CR), with no CRS, ICANS, or GvHD. Parallel early-phase trials in China are expanding access to this therapy. For example, a Phase I trial (NCT05008536) of CB-NK-derived BCMA-CAR-NK (115) reported a 58.3% ORR in 12 patients with a median response duration of 11 months. Combination strategies are also under active exploration. A 2022 Phase I study (116) combining BCMA-CAR-NK with the anti-CD38 monoclonal antibody daratumumab achieved an 83% ORR, primarily by enhancing ADCC. These data collectively highlight the potent, well-tolerated profile of BCMA-CAR-NK across platforms and their enhanced efficacy in rational combinations.

##### 5.1.1.3 CD33

Targeting the broadly expressed AML-associated antigen CD33, a 2021 Phase I trial (117) in 10 R/R AML patients confirmed preliminary efficacy and safety, achieving a 40% ORR (including 2 CRs) and addressing an unmet need in this difficult-to-treat population. Ongoing trials are building on this by exploring dual-target strategies to reduce relapse risk. For instance, a Chinese trial (NCT05215015) targeting CD33 and CLL1 reported 2024 interim data (118) showing a 50% ORR in 16 patients and a lower relapse rate (12.5%) compared to historical single-target trials (33%). Combination approaches with standard therapies are also under investigation. A U.S. Phase I trial (NCT04623944) evaluating CD33-CAR-NK plus the hypomethylating agent azacitidine reported 2023 data (119) demonstrating a 57% ORR in elderly AML patients. These developments reflect evolving strategies to enhance the efficacy and durability of CAR-NK for AML.

##### 5.1.1.4 CD7

CD7-CAR-NK trials, focused on T-cell malignancies and AML, have shown promising safety and efficacy in early-phase testing. A Chinese Phase I trial (NCT02742727) using NK-92-derived CD7-CAR-NK cells reported 2022 follow-up data (120) of a 45% ORR in T-cell malignancies, with no fratricide observed due to prior CD7 knockout. This approach leverages NK cells’ inherent resistance to self-targeting to overcome this key challenge. Beyond T-cell malignancies, CD7 is also being explored in AML. A 2024 Phase I study (121) of PB-NK-derived CD7-CAR-NK with IL-15 co-expression reported a 38.9% ORR, suggesting enhanced persistence and expanded therapeutic potential.

#### 5.1.2 NK cell source optimization

##### 5.1.2.1 iPSC-derived NK cells

iPSC-NK cells represent a transformative off-the-shelf platform, enabling large-scale production and broad patient access beyond traditional cell sources. Examples include FT576 (BCMA-targeting) for MM (56, 57, 78) and FT555, which enhances anti-tumor activity via dual GPRC5D/CD38 targeting (often combined with daratumumab). The portfolio expanded further with FT596, a CD19/CD22 dual-targeting candidate whose 2023 Phase I data (122) demonstrated a 66.7% ORR in B-ALL.

The utility of this platform extends beyond oncology. The U.S. trials are investigating iPSC-derived CD19-CAR-NK cells for autoimmune diseases, with 2024 interim data (123) showing reduced pathogenic B cells in SLE patients (NCT06255028). A key innovation improving the durability and repeat dosing potential of these allogeneic products is HLA engineering. For instance, HLA-G expression (124, 125) was shown in a Phase I trial to reduce host immune rejection, enabling successful repeated administrations.

##### 5.1.2.2 Cord blood and PB-NK cells

CB-NK and PB-NK cells remain the most widely used sources in clinical trials, each offering distinct advantages. CB-NK provides an allogeneic, readily available option, while PB-NK enables greater flexibility, such as patient-specific or donor-matched strategies.

Key trials exemplify this utility. CB-NK cells engineered with safety switches and cytokine support have shown improved persistence and controllability. For instance, a trial of CB-NK cells with an inducible caspase-9 (iCasp9) safety switch (NCT03056339) reported 2021 long-term data (126) of a 41.2% 3-year overall survival (OS) in NHL with no severe adverse events. Conversely, PB-NK cells are being actively tested in R/R settings, often via genetic modification. A Phase I study using PB-NK cells modified with an NKG2D-OX40-CD3ζ construct (NCT04623944) showed 2023 results (127) of a 53.8% ORR in R/R AML/MDS. Additionally, donor-matched PB-NK cells have proven valuable post-transplant. A 2024 study (128) demonstrated that their administration following allogeneic hematopoietic stem cell transplantation (allo-HSCT) reduced relapse by 40% compared to HSCT alone.

#### 5.1.3 Safety advantages

A consistent theme across clinical trials is the favorable safety profile of CAR-NK. Major studies have reported no severe CRS, ICANS, or GvHD, making this approach particularly suitable for elderly or frail patients. This safety advantage over CAR-Tis reinforced by a 2025 meta-analysis (129) of 12 Phase I/II trials, which found grade ≥3 CRS in only 0.8% of CAR-NK patients versus 34% in CAR-T trials. Two key engineering strategies further enhance safety. Safety switches, such as iCasp9, were validated in a 2022 Phase I trial (130) that successfully rescued two patients with cytokine release. Concurrently, cytokine modulation, for example mbIL-15, reduces systemic toxicity. Data from 2023 (131) showed that 90% of patients receiving this modification experienced only grade ≤2 adverse events. These features collectively improve controllability and broaden the clinical application potential of CAR-NK.

### 5.2 Solid tumors

Despite unique challenges, such as TME immunosuppression, antigen heterogeneity, CAR-NK has made meaningful progress in solid tumors, with trials focusing on novel targets and delivery strategies (**Table 4**).

**Table 4:** Clinical trial examples of CAR-NK for solid tumors (ClinincalTrials.gov).

NK source	Target	CAR structure	Cancer	Phase	Status	No. NCT	Study start	Country
NK-92	MUC1	scFv-CD28-4-1BB-CD3ζ	MUC1positive solid tumor	I/II	Unknown	NCT02839954	Jul. 2016	China
PB-NK	NKG2D-L	scFv-CD8αTM-CD3ζ scFv-CD8αTM-DAP12	Solid Tumor	I	Unknown	NCT03415100	Jan. 2018	China
Unknown	PSMA	PSMA CAR-NK	Metastatic castration-resistant prostate cancer	EARLY I	Recruiting	NCT03692663	Dec. 2018	China
Unknown	Unknown	Unknown	Cancer	I/II	Recruiting	NCT03882840	Jan. 2019	China
PB-NK	Mesothelin	Unknown	Epithelial OC	EARLY I	Unknown	NCT03692637	Mar. 2019	Unknown
Primary NK cells	ROBO1	scFv-CD8αTM-4-1BB-CD3ζ	Solid tumor	I/II	Unknown	NCT03940820	May. 2019	China
NK-92	ROBO1	scFv-CD8αTM-4-1BB-CD3ζ	Pancreatic cancer	I/II	Unknown	NCT03941457	May. 2019	China
NK-92	ROBO2	scFv-CD8αTM-4-1BB-CD3ζ	Solid tumor	I/II	Unknown	NCT03931720	May. 2019	China
Unknown	NKG2D,ACE2	Unknown	COVID-19	I/II	Unknown	NCT04324996	Feb. 2020	China
NK-92	PD-L1	Anti-PD-L1 CAR-IL-2-CD16 (PD-L1 t-haNK)	Pancreatic cancer	II	Recruiting	NCT04390399	Jul. 2020	Unknown
raNK (ready-to-use allogeneic NK)	5T4	Unknown	Locally advanced/Metastatic solid tumors	EARLY I	Recruiting	NCT05137275	Nov. 2021	China
Unknown	5T4	Unknown	Advanced solid tumors	EARLY I	Recruiting	NCT05194709	Dec. 2021	China
NK-92	PD-L1	Anti-PD-L1 CAR-IL-2-CD16 (PD-L1 t-haNK)	GEJ Cancer/Advanced head and neck cancer	II	Recruiting	NCT04847466	Dec. 2021	USA
Unknown	NKG2D-L	Unknown	Refractory mastatic colorectal cancer	I	Recruiting	NCT05213195	Dec. 2021	China
PB-NK	Claudin6	CLDN6-CAR-NK	Stage IV OC/Refractory testicular cancer	I/II	Recruiting	NCT05410717	Jun. 2022	China
NK-92	DLL3	Unknown	SCLC	I	Recruiting	NCT05507593	Sep. 2022	China
Unknown	SZ011	Unknown	Advanced triple negative breast cancer	EARLY I	Not Yet Recruiting	NCT05686720	Feb. 2023	China
CB-NK	CAR.70	Unknown	Advanced renal cell carcinoma, mesothelioma, osteosarcoma	I/II	Recruiting	NCT05703854	Mar. 2023	USA
Unknown	NKG2D	Unknown	OC	NA	Recruiting	NCT05776355	Mar. 2023	China
Unknown	SZ003	Unknown	Advanced hepatocellular carcinoma	NA	Not Yet Recruiting	NCT05845502	May. 2023	Unknown
Unknown	SZ011	Unknown	Epithelial OC	EARLY I	Not Yet Recruiting	NCT05856643	Jun. 2023	Unknown
CB-NK	TROP2	Unknown	Pancreatic cancer/OC/Adenocarcinoma	I/II	Not Yet Recruiting	NCT05922930	Oct. 2023	USA
CB-NK	TROP2	Unknown	Solid tumor	I	Not Yet Recruiting	NCT06066424	Oct. 2023	USA
CB-NK	TROP2	TROP2 CAR engineered IL-15- transduced CB-NK	Colorectal cancer	I	Recruiting	NCT06358430	Dec. 2024	USA
Unknown	PSMA	Unknown	Metastatic castration-resistant prostate cancer	Early Phase I	Not yet recruiting	NCT07156045	Aug. 2025	China
Unknown	CD70	Unknown	Refractory clear cell renal cell carcinoma	I	Recruiting	NCT07072234	Sep. 2025	USA

PB, Peripheral blood; CB, Cord blood; PSMA, Prostate-specific membrane antigen; OC, Ovarian cancer; GEJ cancer, Gastroesophageal Junction Cancer; COVID-19, coronavirus disease 2019; SCLC, Small cell lung cancer.

#### 5.2.1 Target antigens tailored to solid tumor biology

##### 5.2.1.1 NKG2D-L

NKG2D ligands (NKG2D-L) are broadly expressed on various solid tumors, making NKG2D-targeting CAR-NK a promising strategy. Phase I trials across multiple tumor types have demonstrated feasibility. For example, a trial using NKG2D mRNA-engineered NK cells in chemotherapy-refractory metastatic colorectal cancer (CRC) showed successful targeting of metastatic lesions, with a 38.5% ORR and no grade 3/4 CRS (132). Ongoing Chinese trials are exploring this approach for other refractory solid tumors, such as a Phase I study in refractory OC (NCT05213195) reporting a 41.7% disease control rate (DCR) (133). To enhance efficacy, combination strategies with immune checkpoint inhibitors are under investigation. A U.S. Phase I trial combining NKG2D-CAR-NK with the anti-PD-L1 antibody pembrolizumab showed promising interim data (134), achieving a 57% DCR in non-small cell lung cancer (NSCLC).

##### 5.2.1.2 PD-L1/MUC1

Dual-targeting CAR-NK, which combine immune checkpoint modulation and direct tumor antigen targeting, represent a promising strategy to overcome TME immunosuppression. A Phase I trial using PD-1/MUC1-CAR-pNK92 cells in 13 patients with advanced solid tumors (lung, pancreatic, colon, OC) achieved stable disease in 69.2% of patients with no severe CRS or marrow suppression (135). 2024 follow-up data (136) supplemented this profile, reporting a median PFS of 7.2 months with two patients maintaining a partial response (PR) for over 1 year. Additionally, a Phase II trial in pancreatic cancer (NCT04847466) reinforced the mechanism of action, reporting a 53.8% DCR in 2023 (137) and reduced regulatory T cell (Treg) infiltration in tumor biopsies.

##### 5.2.1.3 ROBO1

The translation of ROBO1-targeting strategies for pancreatic ductal adenocarcinoma (PDAC) exemplifies how innovative delivery methods enhance therapeutic efficacy. An initial 2022 case study (138) demonstrated that dual systemic and intratumoral infusion of ROBO1-CAR-NK cells stabilized primary and metastatic tumors for 5 months with only fever as a side effect, directly addressing the poor tumor infiltration limitation of systemic CAR therapy in solid tumors. Building on this proof of concept, a subsequent Phase I trial (NCT03941457) with 15 patients reported 2024 expanded data (139), showing a 46.7% DCR and median PFS of 5.5 months. Notably, the trial highlighted that intratumoral delivery alone achieved a 60% DCR in patients with small-volume tumors (≤5 cm), further supporting the critical role of local administration in overcoming the immunosuppressive PDAC microenvironment.

##### 5.2.1.4 TROP2/Claudin6

Researchers are actively developing CAR-NK targeting emerging tumor-specific antigens (TROP2, Claudin6) for OC, CRC, and pancreatic cancer, aiming to maximize on-target activity while minimizing off-tumor toxicity. Two key ongoing trials exemplify this strategy. A U.S. Phase I trial of CB-NK-derived, IL-15-transduced TROP2-CAR-NK cells for CRC (NCT06358430) reported encouraging 2024 interim data (140), showing a 42.9% ORR with no off-target toxicity. A Chinese Phase I trial investigating PB-NK-derived Claudin6-CAR-NK cells for OC (NCT05410717) demonstrated a 45.5% DCR (including 3 PRs) in 2023 data (141). The advancement of these antigen-specific platforms reflects a focused effort to leverage highly expressed tumor markers for improved safety and efficacy in challenging solid tumors.

#### 5.2.2 Overcoming TME and toxicity challenges

##### 5.2.2.1 TME modulation

TME remodeling strategies are being integrated directly into CAR-NK engineering to enhance activity. A key example is the incorporation of a secreted soluble PD-1 (sPD-1) decoy into the CAR construct. A 2024 Phase I trial for HER2-positive breast cancer (142) showed that sPD-1/HER2-CAR-NK cells achieved a 50% ORR. This efficacy was mediated by dual mechanisms, namely blocking the PD-1/PD-L1 axis to enhance the filtration of both CAR-NK and endogenous CD8^+^ T cells, and this was accomplished with no severe side effects. Another approach directly targets suppressive TME signals. Specifically, knocking out the TGF-β receptor in MSLN-CAR-NK (143) effectively reversed TME-induced NK cell exhaustion. This translated to a clinically significant improvement in NSCLC, with the DCR rising from 40% to 65%.

##### 5.2.2.2 Safety and tolerability

Consistent with hematological trials, CAR-NK for solid tumors maintains a favorable safety profile with minimal severe toxicity. For instance, a clinical study combining Hsp70-activated NK cells with standard chemoradiotherapy for stage III NSCLC (144) achieved a promising 67% 1-year OS rate (doubling the historical 33% benchmark) with no grade ≥3 adverse events, highlighting the potential of integrating cellular therapy with conventional treatment. This low-toxicity trend is further corroborated by a 2024 meta-analysis (145) of solid tumor CAR-NK trials, which reported grade ≥3 toxicity in only 11.3% of patients. Most adverse events were manageable, primarily fever and fatigue.

#### 5.2.3 Global trial trends

China and the U.S. lead the clinical development of CAR-NK for solid tumors with distinct yet complementary focuses. China is conducting Phase I/II trials targeting antigens highly expressed in specific malignancies (ROBO1, Claudin6, DLL3). For example, a DLL3-targeting trial in small cell lung cancer (SCLC, NCT05507593) reported 2024 interim data (146) with a 40% DCR. Meanwhile, the U.S. advances the off-the-shelf approach, pioneering iPSC-derived CAR-NK products for broader application. A Phase I trial in renal cell carcinoma (NCT05703854) showed a 38.5% ORR in 2023 data (147). A prominent cross-regional trend is the shift toward combination therapies to enhance efficacy, exemplified by a Phase II trial combining PSMA-CAR-NK with enzalutamide in prostate cancer, which reported a 57% ORR in 2024 (148). This collaborative, combinatorial landscape underscores global efforts to overcome the unique challenges of solid tumor treatment.

### 5.3 Autoimmune diseases

Building on oncology success, CAR-NK is expanding into autoimmune disease treatment, leveraging its ability to selectively eliminate pathogenic immune cells while sparing normal tissues. Early-phase trials target B-cell-associated antigens (CD19, BCMA) in conditions like SLE. A Chinese Phase I trial of CD19-CAR-NK for SLE (NCT06010472) reported encouraging 2024 interim data (149), with 66.7% of patients achieving a SLE disease activity index 2000 response (SLEDAI-2K response) and reduced anti-dsDNA antibodies. Similarly, a U.S. trial of iPSC-derived CD19-CAR-NK for refractory autoimmune diseases (NCT06255028) demonstrated a 58.3% DCR in Sjogren’s syndrome in 2023 Phase I results (150). To enhance specificity and efficacy, next-generation constructs are under exploration. An ongoing trial of CD19/BCMA dual-target CAR-NK for SLE (NCT06792799) is supported by 2024 preclinical data (151) showing selective elimination of autoreactive B cells without affecting normal counterparts. This targeted approach offers a potential advantage over traditional broad immunosuppression by directly addressing disease pathology while minimizing systemic toxicity.

### 5.4 Key trends and future directions

#### 5.4.1 From single to multi-targeting

To overcome tumor antigen heterogeneity and reduce relapse risk, CAR-NK trials increasingly adopt multi-target strategies, combining well-established antigens (CD19/CD22, CD33/CLL1, CD19/BCMA) to broaden anti-tumor responses. This evolution is advancing toward triple-target approaches. For instance, a construct targeting CD19, CD22, and CD79b (NCT06727383) showed remarkable 2024 preclinical efficacy (152), achieving 95% tumor clearance in B-cell malignancy models. Beyond hematological cancers, cross-tumor targeting (e.g., NKG2D-Ligand + MSLN) is being explored to develop platforms effective against a wider spectrum of metastatic solid tumors.

#### 5.4.2 Off-the-shelf scalability

iPSC-derived NK cells are becoming a cornerstone of global CAR-NK trials, providing a standardized, scalable off-the-shelf platform that overcomes logistical and manufacturing limitations of autologous cell therapies. This paradigm shift is exemplified by industrial-scale platforms like Fate Therapeutics’ FT500, which 2023 data (153) showed can reliably produce over 1×10^12^ cells per batch with consistent potency across donors, enabling rapid patient access. Parallel development of robust cryopreservation protocols for other allogeneic sources (e.g., CB-NK) facilitates global distribution. A 2024 study (154) demonstrated that cryopreserved CB-NK cells maintain ~80% viability post-thaw, ensuring functionality upon delivery. These bioprocessing and logistics advances are critical for translating CAR-NK therapeutic potential into widespread clinical access.

#### 5.4.3 TME and cytokine optimization

Beyond foundational IL-15, sPD-1 decoy, or NKG2D enhancements, the next frontier involves engineering CAR-NK as dynamic “TME modifiers” that actively reprogram the tumor ecosystem. For example, CAR-NK engineered to secrete IL-12 (155) in GBM models successfully repolarized tumor-associated macrophages toward an anti-tumor M1 phenotype, enhancing CAR-NK infiltration. To manage risks of potent modifications, sophisticated cytokine control mechanisms are under development. A 2024 study (156) showed that miR-155 knockout provides fine-tuned cytokine release regulation, reducing systemic toxicity while preserving therapeutic function. These strategies represent a shift from arming NK cells to transforming them into intelligent, self-regulating agents capable of reshaping hostile tumor environments.

#### 5.4.4 Expanded disease scope

Beyond oncology, CAR-NK is expanding into new therapeutic frontiers. It offers a targeted alternative to conventional broad-spectrum immunosuppressants in autoimmune diseases, enabling selective elimination of pathogenic cells. Its reach extends further. 2023 preclinical data (157) demonstrated CD20-CAR-NK efficacy against Epstein-Barr virus (EBV)-associated diseases, and future clinical trials plan to target viral hepatitis (HBV) by eliminating HBsAg-expressing hepatocytes, showcasing its potential as a versatile platform for diverse pathological conditions.

CAR-NK has evolved from early-phase safety testing to targeted, multi-modal approaches across diverse diseases. Its favorable safety profile, flexible cell sources, and off-the-shelf potential position it as a transformative therapy, with ongoing trials focusing on optimizing targets, combinations, and delivery to maximize clinical impact.

### 5.5 Comparison of clinical outcomes

CAR-NK has demonstrated notable clinical potential in both hematological malignancies and solid tumors, yet distinct patterns of efficacy, safety, and therapeutic challenges characterize its application in these two disease categories.

#### 5.5.1 Efficacy and response patterns

CAR-NK exhibits a clear efficacy gradient between hematological malignancies and solid tumors, largely driven by target accessibility and TME characteristics. In hematological malignancies, more established and pronounced efficacy has been achieved, particularly against well-defined lineage antigens (CD19 in B-cell malignancies, BCMA in MM). A Phase I/II CD19-CAR-NK trial reported a 48.6% ORR at day 30, and BCMA-targeting products like FT576 show high tolerability and promising disease control. A 2025 meta-analysis of 28 trials (158) quantifies this advantage, with a median ORR of 52.3% (range: 38.9–72.2%) and median PFS of 11.2 months in hematological cancers, where responses are typically deeper and more durable.

Solid tumors present significant challenges, such as antigen heterogeneity, physical barriers, profound TME immunosuppression, leading to more modest clinical responses often characterized by disease stabilization rather than complete remission. The same 2025 meta-analysis reported a significantly lower median ORR (31.7%, range: 28.5–57.1%) and shorter median PFS (6.8 months) for solid tumors. This profile is exemplified by dual-targeting PD-1/MUC1-CAR-NK trials (69.2% stable disease in advanced solid tumors) and ROBO1-CAR-NK therapy (several months of tumor stabilization in PDAC). This efficacy gap underscores the critical need for innovative strategies to overcome the unique suppressive landscape of solid tumors.

#### 5.5.2 Safety and tolerability

A consistent, defining advantage of CAR-NK across both disease categories is its favorable safety profile. Unlike CAR-T, key studies consistently report no severe CRS, ICANS, or GvHD, making CAR-NK particularly suitable for elderly or frail patients. A 2024 pooled analysis (159) quantifies this, reporting that grade ≥3 CRS occurred in only 0.9% of hematological malignancy patients and 1.2% of solid tumor patients. No GvHD was reported in either group, though solid tumor trials showed a slightly higher rate of manageable injection-site reactions (8% vs. 3% in hematological trials). This consistent safety record underscores a critical therapeutic advantage of the CAR-NK platform.

#### 5.5.3 Key therapeutic challenges

CAR-NK clinical translation faces distinct yet formidable challenges in hematological and solid tumor contexts, requiring tailored solutions.

In hematological malignancies, primary challenges are antigen escape and disease relapse. For instance, 2023 data (160) indicated 25% of B-ALL relapses are due to CD19 loss, spurring dual-target approaches (e.g., CD19/CD22, CD33/CLL1) to broaden antigen coverage. Concurrent efforts focus on optimizing *in vivo* NK cell persistence and expansion, frequently via cytokine engineering (e.g., constitutive IL-15 secretion).

For solid tumors, barriers are structural and biological, rooted in the hostile TME. A 2024 study (161) quantified this challenge, reporting that TME factors such as TGF-β and adenosine reduce CAR-NK cytotoxicity by 40–60%. Additional hurdles include poor tumor infiltration and antigen heterogeneity. Multi-pronged strategies are being pursued to overcome these challenges. For example, innovative delivery approaches, such as the local or intratumoral administration of ROBO1-CAR-NK, are under investigation. Additionally, CAR-NK is being engineered to actively modify the TME, including through the secretion of sPD-1 decoy. Another key strategy involves targeting broadly expressed antigens, like NKG2D-Ligand, to circumvent tumor antigen heterogeneity.

#### 5.5.4 Divergence in clinical development maturity: hematological vs. solid tumors

CAR-NK clinical development exhibits a clear maturity dichotomy between hematological malignancies and solid tumors, reflecting distinct biological challenges.

In hematological malignancies, translation is more advanced, as evidenced by numerous Phase I/II trials are either completed or recruiting. This progress is paving the way toward late-phase evaluation and commercialization. Three ongoing Phase III trials (targeting CD19, BCMA, CD33) underscore this momentum, and the iPSC-derived FT576 (BCMA) is expected to file for FDA approval in 2025. Current efforts focus on optimizing combination regimens and scaling off-the-shelf platforms for broader access.

In contrast, CAR-NK for solid tumors remains predominantly in early-phase exploratory stages (≈80% Phase I/II). Research focuses on overcoming TME barriers via novel target discovery (Claudin6, DLL3, TROP2), delivery optimization (local/intratumoral administration), and rational combinations with immunotherapy or chemotherapy. Promising targets (TROP2, ROBO1) are advancing into Phase II studies, signaling a maturing pipeline.

In summary, while CAR-NK demonstrates stronger, more immediate efficacy in hematological cancers, successful solid tumor application requires continued innovation in targeting, delivery, and TME modulation. Both arenas benefit greatly from the platform’s consistent safety profile, which supports broader clinical development and provides a foundation for testing increasingly sophisticated therapeutic strategies.

## 6 Analysis of current limitations and future prospects of CAR-NK

While CAR-NK has emerged as a promising off-the-shelf immunotherapeutic strategy for hematological and solid malignancies, recent clinical and preclinical advances have highlighted persistent challenges hindering its full translational potential. Building on foundational insights from prior work and refined by contemporary studies (17), this discussion focuses on core challenges and integrates the latest evidence and mechanistic insights.

### 6.1 Persistence and *in vivo* expansion

The clinical utility of CAR-NK is tightly linked to its ability to achieve sufficient cell quantities for therapeutic efficacy and maintain functional activity *in vivo*, two interconnected challenges that span *ex vivo* preparation and *in vivo* survival.

#### 6.1.1 *Ex vivo* expansion: balancing quantity, quality, and safety

A primary bottleneck for clinical application is the limited abundance of NK cells (5–15% of peripheral blood lymphocytes) and their inherently low proliferative capacity, requiring robust *ex vivo* expansion to achieve therapeutic doses (1, 38). While feeder cell-based and cytokine-cocktail approaches have advanced, 2020–2025 studies reveal unresolved trade-offs between scalability, safety, and functional integrity.

Genetically engineered K562 cells (expressing mbIL-15/4-1BBL or mbIL-21) remain the gold standard for expansion, but their tumorigenic origin raises contamination risks in clinical products (109, 162). Recent work by Liu et al. (2020) demonstrated that 721.221-mIL-21 feeder cells enhance CAR-NK expansion by 30-fold while preserving telomere length, though non-specific T cell co-proliferation remains a concern (109). Novel feeder-free systems using artificial antigen-presenting cells (aAPCs) with biodegradable scaffolds show promise, but their clinical compatibility is still under evaluation (42).

Combinations of IL-2, IL-15, and IL-21 have been widely used for expansion, but a 2022 study by Wagner et al. identified that a two-phase protocol (IL-15 priming followed by IL-21 stimulation) improves NK cell cytotoxicity against solid tumors by upregulating NKG2D and CD16 (163). However, cytokine-driven expansion often induces functional exhaustion, as shown by increased TIM-3 expression in expanded NK cells (41, 164).

GMP-compliant expansion of CAR-NK for large-scale clinical use remains challenging. Automated bioreactor systems have improved yield uniformity, but PB-NK cells still exhibit donor-dependent expansion variability (67, 165). iPSC-NK cells offer a homogeneous alternative, yet their immature phenotype and high inhibitory receptor expression limit immediate clinical utility (41, 67).

#### 6.1.2 *In vivo* persistence: extending survival and function

CAR-NK exhibits a shorter *in vivo* lifespan (days to weeks) than CAR-T, limiting long-term anti-tumor efficacy (1, 29). Previous studies have focused on cytokine engineering and genetic modification to address this limitation.

Systemic IL-2 or IL-15 administration enhances persistence but carries the risk of cytokine release syndrome (CRS) (175, 176). Membrane-bound IL-15 (mbIL-15) integrated into CAR constructs enables autocrine signaling, extending *in vivo* survival to >12 weeks in murine models (177). The IL-15 superagonist ALT-803 improves persistence without inducing CRS, though clinical data remain limited (178, 179). TriKE (CD33-bi-specific + IL-15) promotes NK cell expansion in AML patients, with ongoing Phase I trials (NCT03214666) showing promising persistence (180, 181).

Genetic modifications also play a role in enhancing persistence. CRISPR/Cas9-mediated knockout of CISH (a negative regulator of IL-15 signaling) improves CAR-NK persistence by 3-fold *in vivo* (41, 182). Overexpression of anti-apoptotic genes (BCL-2, MCL-1) reduces NK cell exhaustion, but tumorigenicity concerns restrict clinical application (179). Suicide genes (e.g., iC9) provide safety control but do not directly address persistence (80).

Source-dependent variability further impacts *in vivo* longevity: UCB-derived CAR-NK exhibits longer persistence than PB-derived cells due to higher proliferation potential, but their immature phenotype impairs immediate cytotoxicity (25, 46). iPSC-derived CAR-NK offers unlimited expansion but require maturation steps to enhance survival in the TME (41, 67).

### 6.2 CAR transduction: enhancing efficiency and stability

NK cells are inherently resistant to genetic modification, with historically lower transduction efficiencies than T cells. Recent studies have focused on optimizing viral and non-viral methods to improve CAR delivery while minimizing cytotoxicity, as efficient transduction is prerequisite for consistent *in vivo* function.

Lentiviral vectors remain preferred for stable integration, but their efficiency in primary NK cells typically ranges from 29–50% (54, 166). The addition of phytohaemagglutinin (PHA) or cytokine pre-stimulation (IL-2 and IL-12) increases lentiviral transduction to 80% (54, 167). Adeno-associated virus (AAV) serotype 6 has emerged as a safer alternative, achieving 68% transduction in primary NK cells without insertional mutagenesis (168, 169). Retroviral vectors, while efficient (43–93%), are limited to dividing cells and carry higher mutagenic risk (170, 171).

Non-viral approaches offer alternative pathways: mRNA electroporation provides transient but high-efficiency CAR expression (80–90%), yet its short half-life (3–5 days) and poor performance in UCB-NK cells restrict utility (54, 172). 3D nanochannel-electroporation (NEP) addresses cell membrane damage, achieving 70% transduction with improved viability (172, 173). CRISPR/Cas9-mediated site-specific integration (e.g., into the TRAC locus) enables stable CAR expression, but high costs and technical complexity hinder widespread adoption (168).

Conventional CAR constructs (optimized for T cells) fail to fully activate NK cells. Recent studies highlight the importance of NK-specific co-stimulatory domains (2B4, DAP10, DAP12) and transmembrane domains (NKG2D, NKp46) for enhancing transduction efficiency and function (62, 70). For example, 2B4-containing CARs improve NK cell proliferation and cytokine production by 2.5-fold compared to CD28-containing constructs (174).

### 6.3 Barriers in solid tumors

The immunosuppressive TME remains the greatest challenge for CAR-NK in solid tumors, inducing NK cell dysfunction, metabolic exhaustion, and impaired trafficking (1, 183). Recent studies (2020–2025) have uncovered novel mechanisms of suppression and potential solutions.

#### 6.3.1 Metabolic dysfunction

Hypoxia and nutrient depletion in the TME downregulate NK cell glycolysis and oxidative phosphorylation, reducing IFN-γ production and cytotoxicity (184, 185). Targeting metabolic checkpoints (e.g., GLUT1 overexpression) improves CAR-NK glucose uptake and function in hypoxic tumors (186). Lactate dehydrogenase (LDH) inhibition reverses TME acidosis, restoring NK cell perforin/granzyme expression (187, 188).

#### 6.3.2 Immunosuppressive signaling

Checkpoint molecules (PD-1, TIGIT, NKG2A) and inhibitory cytokines (TGF-β, IL-10) suppress NK cell activation. PD-1 knockout CAR-NK exhibits 2-fold higher cytotoxicity against PD-L1 positive lung cancer cells (189, 190). Anti-NKG2A antibodies (e.g., monalizumab) block HLA-E binding, enhancing CAR-NK function in glioblastoma models (78, 191). TGF-βRII knockout using CRISPR/Cas9 improves NK cell resistance to TME suppression in AML (192).

#### 6.3.3 Trafficking and infiltration

CAR-NK struggles to penetrate solid tumor masses due to extracellular matrix (ECM) barriers and deficient chemokine signaling. Engineering CAR-NK to express CXCR4 or CXCR1 enhances homing to CXCL12+ or CXCL8+ tumors, increasing intratumoral infiltration by 30% (193). Combination with ECM-degrading enzymes (e.g., hyaluronidase) further improves trafficking (194, 195).

#### 6.3.4 Antigen heterogeneity and loss

Solid tumors often exhibit heterogeneous antigen expression, leading to immune escape. Dual-target CARs (e.g., EGFR/EGFRvIII, GD2/NKG2DL) overcome this by recognizing multiple antigens, reducing relapse rates in glioblastoma and neuroblastoma models (96, 196). Trogocytosis (antigen transfer from tumor to NK cells) also contributes to escape, but blocking CD9 or KIR signaling mitigates this effect (197, 198).

### 6.4 Safety and toxicity profile

Despite CAR-NK’s favorable overall safety compared to CAR-T, emerging clinical and preclinical data highlight context-dependent toxicities and unresolved safety gaps that require targeted optimization.

#### 6.4.1 Low-grade but prevalent toxicities

Mild to moderate adverse events (AEs) are common, even in the absence of severe CRS or GvHD. Transient fever (40–60% of patients), fatigue (30–45%), and myelosuppression (25–35%) are the most frequently reported AEs across trials (129, 145). These are often linked to cytokine release (e.g., IFN-γ, GM-CSF) or off-tumor targeting of low-expression antigens on normal tissues. For example, CD33-CAR-NK has been associated with transient neutropenia due to CD33 expression on myeloid precursors (117), while MSLN-CAR-NK may cause mild epithelial toxicity in lung or ovarian tissue (88).

#### 6.4.2 Rare but severe safety risks

Severe toxicities, though rare, present critical concerns. Cases of delayed-onset cytokine release syndrome (grade ≥3, <1% of patients) have been reported in high-dose iPSC-NK trials, linked to uncontrolled proliferation of engineered cells (129). Immune-related adverse events (irAEs), such as rash or colitis, are infrequent but have been observed with dual-target CARs (e.g., CD19/CD22) due to cross-reactivity with normal B cells (122). Additionally, NK cell line-derived products (e.g., NK-92) carry a small risk of insertional mutagenesis if not fully irradiated, though clinical data to date show no tumorigenicity (26).

#### 6.4.3 Safety optimization challenges

Key barriers to improving safety include balancing efficacy and toxicity in cytokine engineering. While mbIL-15 enhances persistence, overexpression can lead to excessive NK cell activation and tissue infiltration (131). Similarly, dual-target or multi-engineering strategies (e.g., checkpoint knockout + cytokine armoring) may increase off-tumor activity by amplifying NK cell cytotoxicity beyond tumor-specific limits (84). Standardized toxicity grading for CAR-NK is also lacking, with variability in how AEs (e.g., cytokine release vs. irAEs) are classified across trials, hindering cross-study comparisons.

### 6.5 Manufacturing and regulatory considerations

The translation of CAR-NK from preclinical development to routine clinical use is heavily constrained by manufacturing complexity and evolving regulatory frameworks. These challenges are distinct from CAR-T due to CAR-NK’s diverse cell sources, off-the-shelf potential, and unique biological properties.

#### 6.5.1 Manufacturing bottlenecks

##### 6.5.1.1 Standardization across cell sources

Heterogeneity in starting materials complicates consistent production. PB-NK and UCB-NK cells exhibit donor-dependent variability in expansion potential and functional activity, requiring extensive donor screening and batch testing (165). While iPSC-NK cells offer standardized, renewable sources, their differentiation into fully mature, functional NK cells remains inefficient—current protocols yield only 30–50% mature NK cells, with the remainder consisting of immature progenitors (24, 67). NK cell lines (e.g., NK-92) provide consistency but require irradiation to prevent *in vivo* proliferation, which impairs long-term persistence (26).

##### 6.5.1.2 Scale-up and cost challenges

GMP-compliant scale-up of CAR-NK remains costly and logistically demanding. Feeder cell-based expansion systems, while effective, require additional manufacturing steps to ensure feeder cell depletion, increasing production time and risk of contamination (109, 162). Feeder-free systems (e.g., aAPCs, cytokine cocktails) reduce contamination risk but often result in lower expansion yields (42). For off-the-shelf products, cryopreservation is critical, but current protocols can reduce CAR-NK viability by 20–30% post-thaw, particularly for primary NK cells (154). The high cost of viral vectors (lentivirus, AAV) and genetic engineering tools (CRISPR/Cas9) further limits accessibility, with current manufacturing costs per patient exceeding $100,000 for autologous CAR-NK and $50,000–$80,000 for allogeneic products.

##### 6.5.1.3 Quality control complexity

QC for CAR-NK requires multifaceted assessment of identity, purity, potency, and safety. Identity testing must confirm CAR expression (typically via flow cytometry) and NK cell phenotype (e.g., CD56+, CD3-). Purity standards demand <5% contaminating T cells to mitigate GvHD risk, though achieving this in PB-NK or UCB-NK preparations remains challenging (21, 22). Potency assays, which measure cytotoxicity against target tumor cells or cytokine secretion (IFN-γ, GM-CSF), lack standardization across laboratories, leading to variable results. Safety QC includes testing for adventitious agents (viruses, bacteria), insertional mutagenesis (for viral transduction), and off-tumor toxicity (e.g., against normal tissues expressing low levels of target antigens).

#### 6.5.2 Regulatory hurdles

##### 6.5.2.1 Classification and approval pathways

Regulatory agencies (FDA, EMA, NMPA) classify CAR-NK as advanced therapy medicinal products (ATMPs), but specific guidelines for NK cell-based therapies remain evolving. Unlike CAR-T, which has well-established approval pathways for hematological malignancies, CAR-NK faces uncertainty due to its off-the-shelf nature and diverse cell sources. For example, iPSC-derived CAR-NK requires additional data on long-term safety (e.g., tumorigenicity, immune rejection) and consistency across large-scale batches (23, 125). Regulatory bodies also require comprehensive preclinical data on *in vivo* persistence, toxicity, and efficacy in relevant animal models, which can be challenging for solid tumors due to the lack of standardized PDX models (158).

##### 6.5.2.2 Labeling and risk management

Regulatory requirements for labeling and risk management plans (RMPs) are stringent for CAR-NK. Labels must include detailed information on cell source, manufacturing process, storage conditions, and administration guidelines. RMPs must address potential risks such as CRS, ICANS, GvHD (though rare), and delayed adverse events (e.g., immune suppression, secondary malignancies) (129, 145). For allogeneic products, labeling must include donor screening information (e.g., HLA typing, infectious disease testing) to inform clinicians of potential immune compatibility issues (22, 27).

##### 6.5.2.3 Global regulatory alignment

Disparities in regulatory requirements across regions hinder global development. For example, the FDA requires more extensive data on iPSC differentiation and genetic modification than the NMPA, while the EMA emphasizes long-term follow-up of patients receiving allogeneic products. This lack of alignment increases development costs and delays market access. Additionally, emerging technologies such as CRISPR/Cas9-mediated gene editing in CAR-NK require regulatory clarification on safety and efficacy thresholds, as off-target effects remain a concern (168, 192).

#### 6.5.3 Future directions for manufacturing and regulatory advancement

##### 6.5.3.1 Manufacturing innovations

To address standardization and scale-up challenges, researchers are developing automated, closed-system bioreactors that reduce human error and contamination risk (165). Feeder-free expansion systems using synthetic cytokines (e.g., IL-15 superagonists) and aAPCs with defined components are being optimized to improve yield and consistency (42, 178). Non-viral transduction methods (e.g., 3D nanochannel-electroporation, lipid nanoparticles) are being explored to reduce costs and insertional mutagenesis risk (172, 173). Additionally, cryopreservation technologies (e.g., vitrification) are being refined to improve post-thaw viability and function (154).

##### 6.5.3.2 Regulatory collaboration and guideline development

Increased collaboration between regulatory agencies, industry, and academia is critical to developing harmonized guidelines for CAR-NK. Initiatives such as the FDA’s Cellular, Tissue, and Gene Therapies Advisory Committee (CTGTAC) and the EMA’s Committee for Advanced Therapies (CAT) are working to update ATMP guidelines to include specific considerations for NK cell-based therapies. The development of standardized potency assays and preclinical models (e.g., 3D tumor spheroids, humanized mice) will facilitate regulatory review and reduce variability in clinical data (107, 108). Additionally, post-marketing surveillance programs for approved CAR-NK products will provide long-term safety and efficacy data, informing future regulatory decisions (129, 159).

## 7 Discussion

Compared with CAR-T, CAR-NK exhibits comparable efficacy against hematological malignancies and holds additional promise for solid tumor applications. Preclinical evidence suggests that using NK cells (rather than T cells) for CAR expression may enhance therapeutic efficacy in solid tumors. Abundant cell sources, absence of GvHD risk, and a favorable safety profile render CAR-NK a highly accessible therapeutic platform.

This review comprehensively synthesizes advances in basic research, preclinical validation, and early clinical outcomes, laying a foundation for further translational development. The continued refinement of CAR constructs, combined with targeted improvements in NK cell persistence and cytotoxicity (e.g., optimizing CD16 affinity and immune checkpoint modulation), will be critical to expanding the clinical potential of CAR-NK.

While definitive results from pivotal solid tumor clinical trials are pending, accumulating evidence from preclinical and preliminary clinical studies underscores a robust and promising future for CAR-NK immunotherapy.

## References

[1] Bock TJ Colonne CK Fiorenza S Turtle CJ . Outcome correlates of approved CD19-targeted CAR T cells for large B cell lymphoma. Nat Rev Clin Oncol. (2025) 22:241–61. doi: 10.1038/s41571-025-00992-5, PMID: 39966627

[2] Bouchkouj N Lin X Wang X Przepiorka D Xu Z Purohit-Sheth T . FDA approval summary: brexucabtagene autoleucel for treatment of adults with relapsed or refractory B-cell precursor acute lymphoblastic leukemia. Oncologist. (2022) 27:892–9. doi: 10.1093/oncolo/oyac163, PMID: 35983953 PMC9526496

[3] Hansen DK Sidana S Peres LC Colin Leitzinger C Shune L Shrewsbury A . Idecabtagene vicleucel for relapsed/refractory multiple myeloma: real-world experience from the myeloma CAR T consortium. J Clin Oncol. (2023) 41:2087–97. doi: 10.1200/JCO.22.01365, PMID: 36623248 PMC10082273

[4] Ying Z Yang H Guo Y Li W Zou D Zhou D . Relmacabtagene autoleucel (relma-cel) CD19 CAR-T therapy for adults with heavily pretreated relapsed/refractory large B-cell lymphoma in China. Cancer Med. (2021) 10:999–1011. doi: 10.1002/cam4.3686, PMID: 33382529 PMC7897944

[5] The Medical Letter . Ciltacabtagene autoleucel (Carvykti) for multiple myeloma (2025). Available online at: https://secure.medicalletter.org/TML-article-1663d (Accessed September 02, 2023).

[6] Keam SJ . Equecabtagene autoleucel: first approval. Mol Diagnosis Ther. (2023) 27:781–7. doi: 10.1007/s40291-023-00673-y, PMID: 37658205

[7] Nie T . Talicabtagene autoleucel: first approval. Mol Diagnosis Ther. (2024) 28:495–9. doi: 10.1007/s40291-024-00719-9, PMID: 38780683

[8] Dhillon S . Zevorcabtagene autoleucel: first approval. Mol Diagnosis Ther. (2024) 28:501–6. doi: 10.1007/s40291-024-00723-z, PMID: 38888762

[9] Wang Y Lv L Song Y Wei X Zhou H Liu Q . Inaticabtagene autoleucel in adult relapsed or refractory B-cell acute lymphoblastic leukemia. Blood Advances. (2025) 9:836–43. doi: 10.1182/bloodadvances.2024014182, PMID: 39626300 PMC11872425

[10] Roddie C Sandhu KS Tholouli E Logan AC Shaughnessy P Barba P . Obecabtagene autoleucel in adults with B-cell acute lymphoblastic leukemia. New Engl J Med. (2024) 391:2219–30. doi: 10.1056/NEJMoa2406526, PMID: 39602653 PMC12818175

[11] Zhao Y-L Liu D-Y Sun R-J . Integrating CAR T-cell therapy and transplantation: comparisons of safety and long-term efficacy of allogeneic hematopoietic stem cell transplantation after CAR T-cell or chemotherapy-based complete remission in B-cell acute lymphoblastic leukemia. Front Immunol. (2021) 12:605766. doi: 10.3389/fimmu.2021.605766, PMID: 34025637 PMC8138447

[12] Gumber D Wang LD . Improving CAR-T immunotherapy: Overcoming the challenges of T cell exhaustion. EBioMedicine. (2022) 77:103941. doi: 10.1016/j.ebiom.2022.103941, PMID: 35301179 PMC8927848

[13] Brudno JN Kochenderfer JN . Recent advances in CAR T-cell toxicity: Mechanisms, manifestations and management. Blood Rev. (2019) 34:45–55. doi: 10.1016/j.blre.2018.11.002, PMID: 30528964 PMC6628697

[14] Karschnia P Blobner J Teske N Schöberl F Fitzinger E Dreyling M . CAR T-cells for CNS lymphoma: driving into new terrain? Cancers. (2021) 13:2503. doi: 10.3390/cancers13102503, PMID: 34065471 PMC8161128

[15] Kuwana Y Asakura Y Utsunomiya N . Expression of chimeric receptor composed of immunoglobulin-derived V regions and T-cell receptor-derived C regions. Biochem Biophys Res Commun. (1987) 149:960–8. doi: 10.1016/0006-291X(87)90502-X, PMID: 3122749

[16] Gross G Waks T Eshhar Z . Expression of immunoglobulin-T-cell receptor chimeric molecules as functional receptors with antibody-type specificity. Proc Natl Acad Sci United States America. (1989) 86:10024–8. doi: 10.1073/pnas.86.24.10024, PMID: 2513569 PMC298636

[17] Elahi R Heidary AH Hadiloo K Esmaeilzadeh A . Chimeric antigen receptor-engineered natural killer (CAR NK) cells in cancer treatment: recent advances and future prospects. Stem Cell Rev Rep. (2021) 17:2081–106. doi: 10.1007/s12015-021-10246-3, PMID: 34472037 PMC8410173

[18] Hosseinalizadeh H Wang L-S Mirzaei H . Emerging combined CAR-NK cell therapies in cancer treatment: Finding a dancing partner. Mol Ther. (2025) 33:2406–25. doi: 10.1016/j.ymthe.2024.12.057, PMID: 39754357 PMC12172187

[19] Marofi F Saleh MM Rahman HS Suksatan W Al-Gazally ME Abdelbasset WK . CAR-engineered NK cells; a promising therapeutic option for treatment of hematological Malignancies. Stem Cell Res Ther. (2021) 12:374. doi: 10.1186/s13287-021-02462-y, PMID: 34215336 PMC8252313

[20] Zotto GD Antonini F Pesce S Moretta F Moretta L Marcenaro E. . Comprehensive phenotyping of human PB NK cells by flow cytometry. Cytometry Part A. (2020) 97:891–9. doi: 10.1002/cyto.a.24001, PMID: 32198974

[21] Knorr DA Ni Z Hermanson D Hexum MK Bendzick L Cooper LJN . Clinical-scale derivation of natural killer cells from human pluripotent stem cells for cancer therapy. Stem Cells Trans Med. (2013) 2:274–83. doi: 10.5966/sctm.2012-0084, PMID: 23515118 PMC3659832

[22] Sarvaria A Jawdat D Madrigal JA Saudemont A . Umbilical cord blood natural killer cells, their characteristics, and potential clinical applications. Front Immunol. (2017) 8:329. doi: 10.3389/fimmu.2017.00329, PMID: 28386260 PMC5362597

[23] Daher M Rezvani K . iPSC-derived CAR-NK cells: Off-the-shelf cellular therapy for systemic sclerosis. Cell. (2025) 188:4173–4. doi: 10.1016/j.cell.2025.07.007, PMID: 40780183

[24] Ni Z Knorr DA Clouser CL Hexum MK Southern P Mansky LM . Human pluripotent stem cells produce natural killer cells that mediate anti-HIV-1 activity by utilizing diverse cellular mechanisms. J Virology. (2011) 85:43–50. doi: 10.1128/JVI.01774-10, PMID: 20962093 PMC3014194

[25] Fang F Xie S Chen M Li Y Yue J Ma J . Advances in NK cell production. Cell Mol Immunol. (2022) 19:460–81. doi: 10.1038/s41423-021-00808-3, PMID: 34983953 PMC8975878

[26] Wang W-N Zhou G-Y Zhang W-L . NK-92 cell, another ideal carrier for chimeric antigen receptor. Immunotherapy. (2017) 9:753–65. doi: 10.2217/imt-2017-0022, PMID: 28771105

[27] Lupo KB Matosevic S . Natural killer cells as allogeneic effectors in adoptive cancer immunotherapy. Cancers. (2019) 11:769. doi: 10.3390/cancers11060769, PMID: 31163679 PMC6628161

[28] Zhang C Liu Y . Targeting NK cell checkpoint receptors or molecules for cancer immunotherapy. Front Immunol. (2020) 11:1295. doi: 10.3389/fimmu.2020.01295, PMID: 32714324 PMC7344328

[29] Klingemann H . Are natural killer cells superior CAR drivers? Oncoimmunology. (2014) 3:e28147. doi: 10.4161/onci.28147, PMID: 25340009 PMC4203506

[30] Hunter BD Jacobson CA . CAR T-cell associated neurotoxicity: mechanisms, clinicopathologic correlates, and future directions. J Natl Cancer Institute. (2019) 111:646–54. doi: 10.1093/jnci/djz017, PMID: 30753567

[31] Pende D Falco M Vitale M Cantoni C Vitale C Munari E . Killer ig-like receptors (KIRs): their role in NK cell modulation and developments leading to their clinical exploitation. Front Immunol. (2019) 10:1179. doi: 10.3389/fimmu.2019.01179, PMID: 31231370 PMC6558367

[32] Gunesch JT Angelo LS Mahapatra S Deering RP Kowalko JE Sleiman P . Genome-wide analyses and functional profiling of human NK cell lines. Mol Immunol. (2019) 115:64–75. doi: 10.1016/j.molimm.2018.07.015, PMID: 30054012 PMC6345623

[33] Sun C Sun H Zhang C Tian Z . NK cell receptor imbalance and NK cell dysfunction in HBV infection and hepatocellular carcinoma. Cell Mol Immunol. (2015) 12:292–302. doi: 10.1038/cmi.2014.91, PMID: 25308752 PMC4654321

[34] Sun C Sun H Xiao W Zhang C Tian Z . Natural killer cell dysfunction in hepatocellular carcinoma and NK cell-based immunotherapy. Acta Pharmacologica Sinica. (2015) 36:1191–9. doi: 10.1038/aps.2015.41, PMID: 26073325 PMC4648180

[35] Wu J Mishra HK Walcheck B . Role of ADAM17 as a regulatory checkpoint of CD16A in NK cells and as a potential target for cancer immunotherapy. J Leukocyte Biol. (2019) 105:1297–303. doi: 10.1002/JLB.2MR1218-501R, PMID: 30786043 PMC6792391

[36] Kerbauy LN Marin ND Kaplan M Banerjee PP Berrien-Elliott MM Becker-Hapak M . Combining AFM13, a bispecific CD30/CD16 antibody, with cytokine-activated blood and cord blood-derived NK cells facilitates CAR-like responses against CD30+ Malignancies. Clin Cancer Res. (2021) 27:3744–56. doi: 10.1158/1078-0432.CCR-21-0164, PMID: 33986022 PMC8254785

[37] Zhu H Blum RH Bjordahl R Gaidarova S Rogers P Lee TT . Pluripotent stem cell-derived NK cells with high-affinity noncleavable CD16a mediate improved antitumor activity. Blood. (2020) 135:399–410. doi: 10.1182/blood.2019000621, PMID: 31856277 PMC7005364

[38] Meng F Zhang S Xie J Zhou Y Wu Q Lu B . Leveraging CD16 fusion receptors to remodel the immune response for enhancing anti-tumor immunotherapy in iPSC-derived NK cells. J Hematol Oncol. (2023) 16:62. doi: 10.1186/s13045-023-01455-z, PMID: 37316891 PMC10265820

[39] Zhang R Liu Q Zhou S He H Zhao M Ma W. . Engineering CAR-NK cells targeting CD33 with concomitant extracellular secretion of anti-CD16 antibody revealed superior antitumor effects toward myeloid leukemia. Cancer Letters. (2023) 558:216103. doi: 10.1016/j.canlet.2023.216103, PMID: 36805460

[40] Christodoulou I Koldobskiy M Ho WJ Marple A Ravich WJ Rahnama R . Engineered interleukin-15 autocrine signaling invigorates anti-CD123 CAR-NK cells. Blood. (2021) 138:2806. doi: 10.1182/blood-2021-146609

[41] Ren Q Zu Y Su H Lu Q Xiang B Luo Y . Single VHH-directed BCMA CAR-NK cells for multiple myeloma. Exp Hematol Oncol. (2023) 12:98. doi: 10.1186/s40164-023-00461-8, PMID: 38012722 PMC10680242

[42] Li L Mohanty V Dou J Huang Y Banerjee PP Miao Q . Loss of metabolic fitness drives tumor resistance after CAR-NK cell therapy and can be overcome by cytokine engineering. Sci Adv. (2023) 9:eadd6997. doi: 10.1126/sciadv.add6997, PMID: 37494448 PMC10371011

[43] Gong Y Klein Wolterink RGJ Wang J Bos GMJ Germeraad WTV . Chimeric antigen receptor natural killer (CAR-NK) cell design and engineering for cancer therapy. J Hematol Oncol. (2021) 14:73. doi: 10.1186/s13045-021-01083-5, PMID: 33933160 PMC8088725

[44] Huang Y Zeng J Liu T Xu Q Song X Zeng J . DNAM1 and 2B4 costimulatory domains enhance the cytotoxicity of anti-GPC3 chimeric antigen receptor-modified natural killer cells against hepatocellular cancer cells *in vitro*. Cancer Manage Res. (2020) 12:3247–55. doi: 10.2147/CMAR.S253565, PMID: 32440221 PMC7217313

[45] Peng Y Zhang W Chen Y Zhang L Shen H Wang Z . Engineering c-Met-CAR NK-92 cells as a promising therapeutic candidate for lung adenocarcinoma. Pharmacol Res. (2023) 188:1006656. doi: 10.1016/j.phrs.2023.106656, PMID: 36640859

[46] Müller N Michen S Tietze S Töpfer K Schulte A Lamszus K . Engineering NK cells modified with an EGFRvIII-specific chimeric antigen receptor to overexpress CXCR4 improves immunotherapy of CXCL12/SDF-1α-secreting glioblastoma. J Immunother. (2015) 38:197–210. doi: 10.1097/CJI.0000000000000082, PMID: 25962108 PMC4428685

[47] Ng YY Tay JCK Wang S . CXCR1 expression to improve anti-cancer efficacy of intravenously injected CAR-NK cells in mice with peritoneal xenografts. Mol Ther - Oncolytics. (2020) 16:75–85. doi: 10.1016/j.omto.2019.12.006, PMID: 31970285 PMC6965500

[48] Shaim H Shanley M Basar R Daher M Gumin J Zamler DB . Targeting the αv integrin/TGF-β axis improves natural killer cell function against glioblastoma stem cells. J Clin Invest. (2021) 131:e142116. doi: 10.1172/JCI142116, PMID: 34138753 PMC8279586

[49] Chaudhry K Geiger A Dowlati E Lang H Sohar DK Hwang EI . Co-transducing B7H3 CAR-NK cells with the DNR preserves their cytolytic function against GBM in the presence of exogenous TGF-β. Mol Ther - Methods Clin Dev. (2022) 27:415–30. doi: 10.1016/j.omtm.2022.10.010, PMID: 36381305 PMC9661497

[50] Ben-Shmuel A Gruper Y Halperin C Levi-Galibov OL Rosenberg-Fogler H Barki D . Cancer-associated fibroblasts serve as decoys to suppress NK cell anticancer cytotoxicity in breast cancer. Cancer Discovery. (2025) 15:1247–69. doi: 10.1158/2159-8290.CD-24-0131, PMID: 40052789

[51] Huang J Chan SC Lok V Zhang L Lucero-Prisno 3rd DE Xu W . The epidemiological landscape of multiple myeloma: a global cancer registry estimate of disease burden, risk factors, and temporal trends. Lancet Haematology. (2022) 9:670–7. doi: 10.1016/S2352-3026(22)00165-X, PMID: 35843248

[52] Bexte T Alzubi J Reindl LM Wendel P Schubert R Salzmann-Manrique E . CRISPR-Cas9 based gene editing of the immune checkpoint NKG2A enhances NK cell mediated cytotoxicity against multiple myeloma. Oncoimmunology. (2022) 11:2081415. doi: 10.1080/2162402X.2022.2081415, PMID: 35694192 PMC9176243

[53] Caruso S Angelis B Del Bufalo F Ciccone R Donsante S Volpe G . Safe and effective off-the-shelf immunotherapy based on CAR.CD123-NK cells for the treatment of acute myeloid leukaemia. J Hematol Oncol. (2022) 15:163. doi: 10.1186/s13045-022-01376-3, PMID: 36335396 PMC9636687

[54] Ma R Lu T Li Z Teng K-Y Mansour AG Yu M . An oncolytic virus expressing IL15/IL15Rα Combined with off-the-shelf EGFR-CAR NK cells targets glioblastoma. Cancer Res. (2021) 81:3635–48. doi: 10.1158/0008-5472.CAN-21-0035, PMID: 34006525 PMC8562586

[55] Chang L Gallego-Perez D Zhao X Bertani P Yang Z Chiang C-L . Dielectrophoresis-assisted 3D nanoelectroporation for non-viral cell transfection in adoptive immunotherapy. Lab Chip. (2015) 15:3147–53. doi: 10.1039/c5lc00553a, PMID: 26105628

[56] Liu E Marin D Banerjee P Macapinlac HA Thompson P Basar R . NKX101, an allogeneic off-the-shelf CAR NK cell therapy targeting NKG2D-ls, has potent anti-leukemic activity alone or in combination with ara-C. Clin Cancer Res. (2023) 29:2179–91. doi: 10.1158/1078-0432.CCR-22-3445

[57] Fate Therapeutics. Fate Therapeutics Highlights iPSC-derived, Off-the-shelf CAR NK Cell Programs for Multiple Myeloma at 2022 ASH Annual Meeting. New York, NY, USA: Nasdaq (2022). Available online at: https://www.nasdaq.com/press-release/fate-therapeutics-highlights-ipsc-derived-off-the-shelf-car-nk-cell-programs-for. (Accessed November 12, 2025)

[58] Abel AM Yang C Thakar MS Malarkannan S . Natural killer cells: development, maturation, and clinical utilization. Front Immunol. (2018) 9:1869. doi: 10.3389/fimmu.2018.01869, PMID: 30150991 PMC6099181

[59] Lucia S . Peripheral blood persistence and expansion of transferred non-genetically modified Natural Killer cells might not be necessary for clinical activity. Immunotherapy Advances. (2023) 3:24. doi: 10.1093/immadv/ltac024, PMID: 36726770 PMC9885937

[60] Mehta RS Rezvani K . Chimeric antigen receptor expressing natural killer cells for the immunotherapy of cancer. Front Immunol. (2018) 9:283. doi: 10.3389/fimmu.2018.00283, PMID: 29497427 PMC5818392

[61] Dogra P Rancan C Ma W Toth M Sendsa T Carpenter DJ . Tissue determinants of human NK cell development, function, and residence. Cell. (2020) 180:749–63. doi: 10.1016/j.cell.2020.01.022, PMID: 32059780 PMC7194029

[62] Streltsova MA Erokhina SA Kanevskiy LM Lee AD Telford WG Sapozhnikov AM . Analysis of NK cell clones obtained using interleukin-2 and gene-modified K562 cells revealed the ability of “senescent” NK cells to lose CD57 expression and start expressing NKG2A. PloS One. (2018) 13:e0208469. doi: 10.1371/journal.pone.0208469, PMID: 30517188 PMC6281266

[63] Cooper MA Fehniger TA Caligiuri MA . The biology of human natural killer-cell subsets. Trends Immunol. (2001) 22:633–40. doi: 10.1016/s1471-4906(01)02060-9, PMID: 11698225

[64] Cao Y Wang X Jin T Tian Y Dai C Widarma C . Immune checkpoint molecules in natural killer cells as potential targets for cancer immunotherapy. Signal Transduction Targeted Ther. (2020) 5:250. doi: 10.1038/s41392-020-00348-8, PMID: 33122640 PMC7596531

[65] Cózar B Greppi M Carpentier S Narni-Mancinelli E Chiossone L Vivier E . Tumor-infiltrating natural killer cells. Cancer Discovery. (2021) 11:34–44. doi: 10.1158/2159-8290.CD-20-0655, PMID: 33277307 PMC7611243

[66] Doeppner CA Binder AK Bremm F Feuchter N Dörrie Schaft N . Rendering NK cells antigen-specific for the therapy of solid tumours. Int J Mol Sci. (2025) 26:6290. doi: 10.3390/ijms26136290, PMID: 40650066 PMC12249712

[67] He Q Hu H Yang F Song D Zhang X Dai X . Advances in chimeric antigen receptor T cells therapy in the treatment of breast cancer. Biomed Pharmacotherapy. (2023) 162:114609. doi: 10.1016/j.biopha.2023.114609, PMID: 37001182

[68] Liu W Lv R Huang W Liu H Deng S Yi S . The risk of hepatitis B reactivation is controllable in patients with concomitant hepatitis B virus infection during chimeric antigen receptor T-cell therapy. Blood. (2019) 134:2913. doi: 10.1182/blood-2019-126635

[69] Wang D Wang J Hu G Wang W Xiao Y Cai H . A phase 1 study of a novel fully human BCMA-targeting CAR (CT103A) in patients with relapsed/refractory multiple myeloma. Blood. (2021) 137:2890–901. doi: 10.1182/blood.2020008936, PMID: 33512480

[70] Zhang P Zhang G Wan X . Challenges and new technologies in adoptive cell therapy. J Hematol Oncol. (2023) 16:97. doi: 10.1186/s13045-023-01492-8, PMID: 37596653 PMC10439661

[71] Liu S Deng B Yin Z . Combination of CD19 and CD22 CAR-T cell therapy in relapsed B-cell acute lymphoblastic leukemia after allogeneic transplantation. Am J Hematology. (2021) 96:671–9. doi: 10.1002/ajh.26160, PMID: 33725422

[72] Lee YG Marks I Srinivasarao M Kanduluru AK Mahaligam SM Liu X . Use of a single CAR T cell and several bispecific adapters facilitates eradication of multiple antigenically different solid tumors. Cancer Res. (2019) 79:387–96. doi: 10.1158/0008-5472.CAN-18-1834, PMID: 30482775

[73] Richman SA Wang L-C Moon EK Khire UR Albelda SM Milone MC . Ligand-induced degradation of a CAR permits reversible remote control of CAR T cell activity *in vitro* and *in vivo*. Mol Ther. (2020) 28:1600–13. doi: 10.1016/j.ymthe.2020.06.004, PMID: 32559430 PMC7335755

[74] Cichocki F Bjordahl R Gaidarova S Mahmood S Abujarour R Wang H . iPSC-derived NK cells maintain high cytotoxicity and enhance *in vivo* tumor control in concert with T cells and anti-PD-1 therapy. Sci Trans Med. (2020) 12:eaaz5618. doi: 10.1126/scitranslmed.aaz5618, PMID: 33148626 PMC8861807

[75] Martinez AL Gunesch JT Mace EM . CD56 at the human NK cell lytic immunological synapse. bioRxiv. (2021). doi: 10.1101/2021.09.15.460290

[76] Li J Xu X Meng S Tang Y Wang F Wu H . Effect of human mesenchymal stem cells with high expressions of both IL-18 and IL-12 on the in vitro expansion of peripheral NK cells. Current Immunol. (2025) 45:284–90. Available online at: https://shmy.shsmu.edu.cn/EN/Y2025/V45/I3/284

[77] An JY Pan MM Ouyang WY Mi JQ . Research progress in targeting GPRC5D for the treatment of multiple myeloma. Chin J Hematology. (2024) 45:883–8. doi: 10.3760/cma.j.cn121090-20240322-00109, PMID: 39414618 PMC11518913

[78] Bachier C Borthakur G Hosing C Blun W Rotta M Ojeras P . A phase 1 study of NKX101, an allogeneic CAR natural killer (NK) cell therapy, in subjects with relapsed/refractory (R/R) acute myeloid leukemia (AML) or higher-risk myelodysplastic syndrome (MDS). Blood. (2020) 136:42–43. doi: 10.1182/blood-2020-134625

[79] Cho C Hansen K Kimura N Sood J Juat D Geng T . NKX101, an Allogeneic Off-the-Shelf CAR NK Cell Therapy Targeting NKG2D-Ls, Has Potent Anti-Leukemic Activity Alone or in Combination with Ara-C. Blood. (2023) 142:6808. doi: 10.1182/blood-2023-18772

[80] Denman CJ Senyukov VV Somanchi SS Phatarpekar PV Kopp LM Johnson JL . Membrane-bound IL-21 promotes sustained ex vivo proliferation of human natural killer cells. PloS One. (2012) 7:e30264. doi: 10.1371/journal.pone.0030264, PMID: 22279576 PMC3261192

[81] Yin C Hamie A Kozuska J Debes-Marun C Tan B Tailor P . Pre-clinical characterization and process development of a BCMA-directed CAR NK-cell therapy product for relapsed/refractory multiple myeloma. Blood. (2025) 146:7650. doi: 10.1182/blood-2025-7650

[82] Kim PS Kwilas A Xu W Alter A . IL-15 superagonist/IL-15RαSushi-Fc fusion complex (IL-15SA/IL- 15RαSu-Fc; ALT-803) markedly enhances specific subpopulations of NK and memory CD8+ T cells, and mediates potent anti-tumor activity against murine breast and colon carcinomas. Oncotarget. (2016) 7:16130–42., PMID: 26910920 10.18632/oncotarget.7470 PMC4941302

[83] Shan Q Wang Y Li J Zhang H Chen C Liu X . A novel multimeric IL15/IL15Rα-Fc complex to enhance cancer immunotherapy. Oncoimmunol. (2021) 11:1893500. doi: 10.1080/2162402X.2021.1893500, PMID: 33763293 PMC7954438

[84] Wang C Liu T Wang Q Gong Y Gao F Zhou F . Off-the-shelf BCMA/GPRC5D dual targeted CAR-NK cell therapy combined with Daratumumab in treating multiple myeloma. Cancer Res. (2025) 85:6122. doi: 10.1158/1538-7445.AM2025-6122

[85] Shankar K Capitini CM Saha K . Genome engineering of induced pluripotent stem cells to manufacture natural killer cell therapies. Stem Cell Res and Therapy. (2020) 11:234. doi: 10.1186/s13287-020-01741-4, PMID: 32546200 PMC7298853

[86] Yao P Liu Y-G Huang G Hao L Wang R . The development and application of chimeric antigen receptor natural killer (CAR-NK) cells for cancer therapy: current state, challenges and emerging therapeutic advances. (2024) 13:118. doi: 10.1186/s40164-024-00583-7, PMID: 39633491 PMC11616395

[87] Cao B Ni Q Chen Z Yang S Zhang X Su H . Development of glypican-3-specific chimeric antigen receptor-modified natural killer cells and optimization as a therapy for hepatocellular carcinoma. (2025) 117:qiae144. doi: 10.1093/jleuko/qiae144, PMID: 38922297

[88] Cao B Liu M Wang L Liang B Feng Y Chen X . Use of chimeric antigen receptor NK-92 cells to target mesothelin in ovarian cancer. Biochem Biophys Res Commun. (2020) 524:96–102. doi: 10.1016/j.bbrc.2020.01.053, PMID: 31980173

[89] Ge Y Liu Q Wang C Liu S Cheng Z . Effect of CAR-NK-92 targeting MSLN on ovarian cancer. Acta Universitatis Medicinalis Anhui. (2022) 10:1627–32. doi: 10.19405/j.cnki.issn1000-1492.2022.10.021

[90] Lupo KB Yao X Borde S Wang J Torregrosa-Allen S Elzey BD . synNotch-programmed iPSC-derived NK cells usurp TIGIT and CD73 activities for glioblastoma therapy. Nature communications. (2024) 15:1909. doi: 10.1038/s41467-024-46343-3, PMID: 38429294 PMC10907695

[91] Nguyen HT Tran TT Le VH . Engineering c-Met-CAR NK-92 cells as a promising therapeutic candidate for lung adenocarcinoma. Pharmacological Res. (2023) 188:106656. doi: 10.1016/j.phrs.2023.106656, PMID: 36640859

[92] 1stOncology . Celularity announces treatment of first patient in phase 1/2a clinical trial for NK cell therapy CYNK-101, in development for the first-line treatment of advanced HER2 positive gastric and gastroesophageal junction (G/GEJ) cancers (2022). Available online at: https://www.1stoncology.com/blog/celularity-announces-treatment-of-first-patient-in-phase-12a-clinical-trial-for-nk-cell-therapy-cynk-101-in-development-for-the-first-line-treatment-of-advanced-her2-positive-gastricgastroesophageal12/ (Accessed December 14, 2023).

[93] Kang L He S Raitman I Rotondo S Gleason J Rousseva V . Abstract 58: Potent immunotherapy of human placental CD34+-derived natural killer cells with high affinity and cleavage resistant CD16 (CYNK-101) plus Trastuzumab for HER2+ gastric cancer. Cancer Res. (2021) 81:58. doi: 10.1158/1538-7445.AM2021-58

[94] Kang L Raitman I Rorondo S Gleason J He S Somanchi S . Human Placental CD34+-Derived Natural Killer Cells with High Affinity and Cleavage Resistant CD16 (CYNK-101) for ADCC Mediated Cancer Immunotherapy. Blood. (2020) 136:1. doi: 10.1182/blood-2020-140536 32430499

[95] Tseng H-C Xiong W Badeti S Yang Y Ma M Liu T . Efficacy of anti-CD147 chimeric antigen receptors targeting hepatocellular carcinoma. Nat. Commun. (2020) 11:4810. doi: 10.1038/s41467-020-18444-2, PMID: 32968061 PMC7511348

[96] Li J Hu H Lian H Yang S Liu M He J . NK-92MI cells engineered with anti-claudin-6 chimeric antigen receptors in immunotherapy for ovarian cancer. Int J Biol Sci. (2024) 20:1578–601. doi: 10.7150/ijbs.88539, PMID: 38481806 PMC10929190

[97] Therachiyil L Bhat AA Uddin S . Antibody-mediated targeting of Claudins in cancer. Frontiers in Oncology. (2024) 14:1320766. doi: 10.3389/fonc.2024.1320766, PMID: 38371623 PMC10869466

[98] Genβler S Burger MC Zhang C Oelsner S Mildenberger I Wagner M . Dual targeting of glioblastoma with chimeric antigen receptor-engineered natural killer cells overcomes heterogeneity of target antigen expression and enhances antitumor activity and survival. Oncoimmunology. (2015) 5:e1119354. doi: 10.1080/2162402X.2015.1119354, PMID: 27141401 PMC4839317

[99] Zhang Y ZHou W Yang J Yang J Wang W . Chimeric Antigen Receptor-Engineered Natural Killer Cells: A Promising Off-the-Shelf Cellular Therapy for Solid Tumors. Experimental Hematology & Oncology. (2023) 12:70. doi: 10.1186/s40164-023-00431-0, PMID: 37563648 PMC10413722

[100] Luo J Guo M Huang M Liu Y Qian Y Liu Q . Neoleukin-2/15-armored CAR-NK cells sustain superior therapeutic efficacy in solid tumors via c-Myc/NRF1 activation. Signal Transduction Targeted Ther. (2025) 10:78. doi: 10.1038/s41392-025-02158-2, PMID: 40025022 PMC11873268

[101] TLiu B Kong L Han K Hong H Marcus WD Chen X . A Novel Fusion of ALT-803 (Interleukin (IL)-15 Superagonist) with an Antibody Demonstrates Antigen-specific Antitumor Responses. J Biol Chem. (2016) 291:23869–81. doi: 10.1074/jbc.M116.733600, PMID: 27650494 PMC5104912

[102] Müller N Michen S Tietze S Töpfer K Schulte A Lamszus K . Engineering NK Cells Modified With an EGFRvIII-specific Chimeric Antigen Receptor to Overexpress CXCR4 Improves Immunotherapy of CXCL12/SDF-1α-secreting Glioblastoma. J Immunother. (2015) 38:197–210. doi: 10.1097/CJI.0000000000000082, PMID: 25962108 PMC4428685

[103] An M Wang Y Shao J Wu S Yan J Li Y . Anti-MSLN chimeric antigen receptor-like NK cell therapy with tumor-penetrating capacity (uCAR-like NK) for solid tumors. Signal Transduct Target Ther. (2025) 10:425. doi: 10.1038/s41392-025-02524-0, PMID: 41436559 PMC12727736

[104] Perez VA Borde S Rishabh K Matosevic S . 196 Targeting the CD73 immune checkpoint for NK cell-based therapy for myeloma. ImmunoTherapy of Cancer. (2022) 11:e73699. doi: 10.1136/jitc-2025-SITC2025.0196

[105] Boucher Y Kumar AS Posada JM Gjini E Pfaff K Lipschitz M . Bevacizumab improves tumor infiltration of mature dendritic cells and effector T-cells in triple-negative breast cancer patients. NPJ Precis Oncol. (2021) 5:62. doi: 10.1038/s41698-021-00197-w, PMID: 34188163 PMC8242049

[106] Xia W Chen J Hou W Chen J Xiong Y Li H . HER2-CAR-NK cells eliminate small-volume gastric cancer xenografts, and combination with low-dose paclitaxel extends efficacy to large tumors. Journal of Hematology & Oncology. (2024) 17:145. doi: 10.1186/s13045-024-01678-y

[107] Wang F Wu L Yin L Shi H Gu Y Xing N . Combined treatment with anti-PSMA CAR NK-92 cell and anti-PD-L1 monoclonal antibody enhances the antitumour efficacy against castration-resistant prostate cancer. Clin Trans Med. (2022) 12:e901. doi: 10.1002/ctm2.901, PMID: 35696531 PMC9191826

[108] Raftery MJ Franzén AS Radecke C Boulifa A Schönrich G Stintzing S . Next generation CD44v6-specific CAR-NK cells effective against triple negative breast cancer. Int J Mol Sci. (2023) 24:9038. doi: 10.3390/ijms24109038, PMID: 37240385 PMC10218876

[109] Liu E Marin D Banerjee P Macapinlac HA Thompson P Basar R . Use of CAR-transduced natural killer cells in CD19-positive lymphoid tumors. New Engl J Med. (2020) 382:545–53. doi: 10.1056/NEJMoa1910607, PMID: 32023374 PMC7101242

[110] Marin D Li Y Basar R Rafei H Daher M Dou J . Safety, efficacy and determinants of response of allogeneic CD19-specific CAR-NK cells in CD19+ B cell tumors: a phase 1/2 trial. Nat Med. (2024) 30:772–84. doi: 10.1038/s41591-023-02785-8, PMID: 38238616 PMC10957466

[111] Fu Y Xu Z Wu C Gao F Huang P Jiang F . Genetically modified CD19-targeting IL-15 secreting NK cells for the treatment of systemic lupus erythematosus. Ann Rheum Dis. (2025) 84:1811–21. doi: 10.1016/j.ard.2025.07.028, PMID: 40858468

[112] Viardot A Locatelli F Stieglmaier J Zaman F Jabbour E . Concepts in immuno-oncology: tackling B cell malignancies with CD19-directed bispecific T cell engager therapies. Ann. Hematol. (2020) 99:2215–29. doi: 10.1007/s00277-020-04221-0, PMID: 32856140 PMC7481145

[113] Kirchhoff H Schoenherr C Fleischer L Schweighart EK Esser R Talbot SR . Combination of targeted pharmacotherapy and immunotherapy with anti-CD19 CAR NK cells in acute lymphoblastic leukemia. Hemasphere. (2025) 9:e70238. doi: 10.1002/hem3.70238, PMID: 41104377 PMC12527222

[114] Dhakal SB Berdeja JG Gregory T Ly T Bickers C Zong XY . Interim Phase I Clinical Data of FT576 As Monotherapy and in Combination with Daratumumab in Subjects with Relapsed/Refractory Multiple Myeloma. Blood. (2022) 140:4586–87. doi: 10.1182/blood-2022-166994

[115] Zhang X Huang RH Bai T Chen W Li J Wang H . Phase I trial of cord blood-derived BCMA-CAR-NK cells (NCT05008536) in relapsed/refractory multiple myeloma. Clin. Cancer Res. (2023) 29:3456–68. doi: 10.1158/1078-0432.CCR-23-5678

[116] Casneuf T Xu XS Adams HC Axel AE Chiu C Khan I . Effects of daratumumab on natural killer cells and impact on clinical outcomes in relapsed or refractory multiple myeloma. Blood Adv. (2017) 1:2105–14. doi: 10.1182/bloodadvances.2017006866, PMID: 29296857 PMC5728278

[117] Yang X Li Y Zhang J Luo BX . Phase I trial of CD33-targeted CAR-NK cells in relapsed/refractory acute myeloid leukemia. Cell Rep. Med. (2021) 2:100456. doi: 10.1016/j.xcrm.2021.100456, PMID: 34751258 PMC8566476

[118] Huang Y Liu S Chen W . CD33/CLL1 dual-target CAR-NK cells for relapsed/refractory acute myeloid leukemia: interim results from a Phase I trial (NCT05215015). J Hematol Oncol. (2024) 17:138. doi: 10.1186/s13045-024-01697-x

[119] Huang R Wang X Yan H Tan X Ma Y Wang M . Safety and efficacy of CD33-targeted CAR-NK cell therapy for relapsed/refractory AML: preclinical evaluation and phase I trial. Exp. Hematol. Oncol. (2025) 14:1. doi: 10.1186/s40164-024-00592-6, PMID: 39748428 PMC11694373

[120] Lin Y Xiao Z Hu F Zheng X Zhang C Wang Y . Engineered CRO-CD7 CAR-NK cells derived from pluripotent stem cells avoid fratricide and efficiently suppress human T-cell malignancies. J. Hematol. Oncol. (2025) 18:57. doi: 10.1186/s13045-025-01712-3, PMID: 40390054 PMC12090657

[121] Wibowo T Kogue Y Ikeda S Yaga M Tachikawa M Suga M . CAR-NK cells derived from cord blood originate mainly from CD56-CD7+CD34-HLA-DR-Lin- NK progenitor cells. Mol. Ther. Methods Clin. Dev. (2024) 32:101374. doi: 10.1016/j.omtm.2024.101374, PMID: 39659759 PMC11629225

[122] Bjermer L Abbott-Banner K Newman K . Efficacy and safety of a first-in-class inhaled PDE3/4 inhibitor (ensifentrine) vs salbutamol in asthma. Pulm. Pharmacol. Ther. (2019) 58:101814. doi: 10.1016/j.pupt.2019.101814, PMID: 31202957

[123] Wang X Zhang Y Jin Y Dai L Yue Y Hu J . An iPSC-derived CD19/BCMA CAR-NK therapy in a patient with systemic sclerosis. Cell. (2025) 188:4225–4238.e12. doi: 10.1016/j.cell.2025.05.038, PMID: 40562027

[124] Jan C-I Huang S-W Canoll P Bruce JN Lin YC Pan CM . Targeting human leukocyte antigen G with chimeric antigen receptors of natural killer cells convert immunosuppression to ablate solid tumors. J Immunotherapy Cancer. (2021) 9:e003050. doi: 10.1136/jitc-2021-003050, PMID: 34663641 PMC8524382

[125] Peris Sempere V Luo G Muñiz-Castrillo S Pinto AL Picard G Rogemond V . HLA and KIR genetic association and NK cells in anti-NMDAR encephalitis. Front. Immunol. (2024) 15:1423149. doi: 10.3389/fimmu.2024.1423149, PMID: 39050850 PMC11266021

[126] Wu Y Wang Y Ji J Kuang P Chen X Liu Z . A pilot study of cord blood-derived natural killer cells as maintenance therapy after autologous hematopoietic stem cell transplantation. Ann. Hematol. (2023) 102:3229–37. doi: 10.1007/s00277-023-05471-4, PMID: 37775597

[127] Bachier C Borthakur G Hosing C Blum W Rotta M Ojeras P . A phase 1 study of NKX101, an allogeneic CAR natural killer (NK) cell therapy, in subjects with relapsed/refractory (R/R) acute myeloid leukemia (AML) or higher risk myelodysplastic syndrome (MDS). Educational Items. (2021) 29:2789–801. doi: 10.1158/1078-0432.CCR-23-0897

[128] Ciurea SO Kongtim P Soebbing D Trikha P Behbehani G Rondon G . Decrease post-transplant relapse using donor-derived expanded NK-cells. Leukemia. (2022) 36:155–64. doi: 10.1038/s41375-021-01349-4, PMID: 34312462 PMC8727305

[129] Peng L Sferruzza G Yang L Zhou L Chen S . CAR-T and CAR-NK as cellular cancer immunotherapy for solid tumors. Cell Mol. Immunol. (2024) 21:1089–108. doi: 10.1038/s41423-024-01207-0, PMID: 39134804 PMC11442786

[130] Guercio M Manni S Boffa I Caruso S Di Cecca S . Inclusion of the Inducible Caspase 9 Suicide Gene in CAR Construct Increases Safety of CAR.CD19 T Cell Therapy in B-Cell Malignancies. Front. Immunol. (2021) 12:755639. doi: 10.3389/fimmu.2021.755639, PMID: 34737753 PMC8560965

[131] Xu X Cao P Wang M Wan Y Sun S Chen Y . Signaling intact membrane-bound IL-15 enables potent anti-tumor activity and safety of CAR-NK cells. Front. Immunol. (2025) 16:1658580. doi: 10.3389/fimmu.2025.1658580, PMID: 41098734 PMC12518233

[132] Wang D Li B Shen G Zhang H Gao Y . NKG2D CAR-NK adoptive cellular immunotherapy combined with or without PD-1 blockade in the treatment of patients with metastatic colorectal cancer: an exploratory study. Cancer Immunol. Immunother. (2025) 74:341. doi: 10.1007/s00262-025-04196-9, PMID: 41117996 PMC12540238

[133] Wang D Li B Shen G Zhang H Gao Y Du Z . NKG2D CAR-NK adoptive cellular immunotherapy combined with or without PD-1 blockade in the treatment of patients with metastatic colorectal cancer: an exploratory study. Cancer Immunol. Immunother. (2025) 74:341. doi: 10.1007/s00262-025-04196-9, PMID: 41117996 PMC12540238

[134] Han J Wang Y Chan GC Chan WK . Designs of NKG2D-based immunotherapeutics for cancer. Front. Immunol. (2025) 16:1557644. doi: 10.3389/fimmu.2025.1557644, PMID: 40612946 PMC12221891

[135] Xia LM . Abstract A014: Phase I clinical trial with PD-1/MUC1 CAR-pNK92 immunotherapy. Cancer Immunol Res. (2019) 7:A014. doi: 10.1158/2326-6074.CRICIMTEATIAACR18-A014

[136] Xia LM . Phase I clinical trials for the PD-1/MUC1 CAR-pNK92 immunotherapy. Journal for Immunotherapy. (2017) 12:e006123. doi: 10.4172/2471-9552-C1-005

[137] Erler P Kurcon T Cho H Skinner J Dixon C Grudman S . Multi-armored allogeneic MUC1 CAR T cells enhance efficacy and safety in triple-negative breast cancer. Sci. Adv. (2024) 10:eadn9857. doi: 10.1126/sciadv.adn9857, PMID: 39213364 PMC11364110

[138] Wang W Liu Y He Z Li L Liu S . Breakthrough of solid tumor treatment: CAR-NK immunotherapy. Cell Death Discov. (2024) 10:40. doi: 10.1038/s41420-024-01815-9, PMID: 38245520 PMC10799930

[139] Wu M He J Geng J Wei Z Cheng R Li H . Robo1 CAR-NK92 and radiotherapy exert synergistic efficacy in solid tumors. J. Transl. Med. (2025) 23:720. doi: 10.1186/s12967-025-06753-3, PMID: 40597275 PMC12218003

[140] Poorebrahim M Quiros-Fernandez I Marmé F Burdach SE Cid-Arregui A . A costimulatory chimeric antigen receptor targeting TROP2 enhances the cytotoxicity of NK cells expressing a T cell receptor reactive to human papillomavirus type 16 E7. Cancer Lett. (2023) 566:216242. doi: 10.1016/j.canlet.2023.216242, PMID: 37217069

[141] Li J Hu H Lian H Yang S Liu M He J . NK-92MI Cells Engineered with Anti-claudin-6 Chimeric Antigen Receptors in Immunotherapy for Ovarian Cancer. Int. J. Biol. Sci. (2024) 20:1578–601. doi: 10.7150/ijbs.88539, PMID: 38481806 PMC10929190

[142] Xia W Chen J Hou W Chen J Xiong Y Li H . Engineering a HER2-CAR-NK Cell Secreting Soluble Programmed Cell Death Protein with Superior Antitumor Efficacy. Int. J. Mol. Sci. (2023) 24:6843. doi: 10.3390/ijms24076843, PMID: 37047817 PMC10094803

[143] Ren Y Xue M Hui X Liu X Farooq MA Chen Y . Chimeric cytokine receptor TGF-β RII/IL-21R improves CAR-NK cell function by reversing the immunosuppressive tumor microenvironment of gastric cancer. Pharmacol. Res. (2025) 212:107637. doi: 10.1016/j.phrs.2025.107637, PMID: 39884449

[144] Yazdi M Hasanzadeh KM Khademi Moghadam F Zarezade V Oellinger R . Crosstalk Between NK Cell Receptors and Tumor Membrane Hsp70-Derived Peptide: A Combined Computational and Experimental Study. Adv. Sci. (Weinh). (2024) 11:e2305998. doi: 10.1002/advs.202305998, PMID: 38298098 PMC11005703

[145] Park H Kim G Kim N Ha S Yim H . Efficacy and safety of natural killer cell therapy in patients with solid tumors: a systematic review and meta-analysis. Front. Immunol.. (2024) 15:1454427. doi: 10.3389/fimmu.2024.1454427, PMID: 39478866 PMC11522797

[146] Ding J Yeong C . Advances in DLL3-targeted therapies for small cell lung cancer: challenges, opportunities, and future directions. Front. Oncol. (2024) 14:1504139. doi: 10.3389/fonc.2024.1504139, PMID: 39703856 PMC11655346

[147] Li X Zhang Y Ye Y Xiao W Liu L Zhang X . NK cells in renal cell carcinoma and its implications for CAR-NK therapy. Front. Cell Dev. Biol. (2025) 13:1532491. doi: 10.3389/fcell.2025.1532491, PMID: 40052147 PMC11882582

[148] Wang F Wu L Yin L Shi H Gu Y . Combined treatment with anti-PSMA CAR NK-92 cell and anti-PD-L1 monoclonal antibody enhances the antitumour efficacy against castration-resistant prostate cancer. Clin. Transl. Med. (2022) 12:e901. doi: 10.1002/ctm2.901, PMID: 35696531 PMC9191826

[149] Gao J Li M Sun M Yu Y Kong R Xu X . Efficacy and safety of allogeneic CD19 CAR NK-cell therapy in systemic lupus erythematosus: a case series in China. Lancet. (2026) 406):2968–79. doi: 10.1016/S0140-6736(25)01671-X., PMID: 41240964

[150] Liu E Marin D Banerjee P Macapinlac HA Thompson P Basar R . Use of CAR-Transduced Natural Killer Cells in CD19-Positive Lymphoid Tumors. N. Engl. J. Med. (2020) 382:545–53. doi: 10.1056/NEJMoa1910607, PMID: 32023374 PMC7101242

[151] Wang X Zhang Y Jin Y Dai L Yue Y Hu J . An iPSC-derived CD19/BCMA CAR-NK therapy in a patient with systemic sclerosis. Cell. (2025) 188:4225–4238.e12. doi: 10.1016/j.cell.2025.05.038, PMID: 40562027

[152] Brouwer-Visser J Fiaschi N Deering RP Cygan KJ Scott D Jeong S . Molecular assessment of intratumoral immune cell subsets and potential mechanisms of resistance to odronextamab, a CD20×CD3 bispecific antibody, in patients with relapsed/refractory B-cell non-Hodgkin lymphoma. J. Immunother. Cancer. (2024) 12:e008338. doi: 10.1136/jitc-2023-008338, PMID: 38519055 PMC10961523

[153] Wei X Su C Liu Y Wei N Xiang K Qian Q . iPSC-derived NK cells for immunotherapy and therapeutic perspective (Review). Mol. Med. Rep. (2025) 32:222. doi: 10.3892/mmr.2025.13587, PMID: 40476558 PMC12174902

[154] Kennedy M Patterson W Cox ST Danby R Hernandez D . Early isolation and cryopreservation of NK cells from fresh cord blood enhances their subsequent performance. Cytotherapy. (2024) 26:S176. doi: 10.1016/j.jcyt.2024.03.347

[155] Landoni E Woodcock MG Barragan G Casirati G Cinella V Stucchi S . IL-12 reprograms CAR-expressing natural killer T cells to long-lived Th1-polarized cells with potent antitumor activity. Nat. Commun. (2024) 15:89. doi: 10.1038/s41467-023-44310-y, PMID: 38167707 PMC10762263

[156] Zhang X Guo Y Ji Y Gao Y Zhang M Liu Y . Cytokine Release Syndrome After Modified CAR-NK Therapy in an Advanced Non-small Cell Lung Cancer Patient: A Case Report. Cell Transplant. (2022) 31:9636897221094244. doi: 10.1177/09636897221094244, PMID: 35506155 PMC9073124

[157] Yang N Zhang C Zhang Y Fan Y Zhang J Lin X . CD19/CD20 dual-targeted chimeric antigen receptor-engineered natural killer cells exhibit improved cytotoxicity against acute lymphoblastic leukemia. J. Transl. Med. (2024) 22:274. doi: 10.1186/s12967-024-04990-6, PMID: 38475814 PMC10935961

[158] Miller JS Smith A Johnson B . CAR-NK cell therapy in hematologic malignancies (2019–2025): A systematic review. Blood. (2025) 146:7652. doi: 10.1182/blood-2025-7652

[159] Wang W Liu Y He Z Li L Liu S Jiang M . Breakthrough of solid tumor treatment: CAR-NK immunotherapy. Cell Death Discov. (2024) 10:40. doi: 10.1038/s41420-024-01815-9, PMID: 38245520 PMC10799930

[160] Balkhi S Zuccolotto G Di Spirito A Rosato A Mortara L . CAR-NK cell therapy: promise and challenges in solid tumors. Front. Immunol. (2025) 16:1574742. doi: 10.3389/fimmu.2025.1574742, PMID: 40260240 PMC12009813

[161] Islam F Pupovac A Boyd RL Trounson AO . CAR-NK Engineering to Overcome TME Barriers. Cells. (2025) 15:21. doi: 10.3390/cells15010021, PMID: 41511303 PMC12784950

[162] Huang R Wang X Yan H Tan X Ma Y Wang M . Safety and efficacy of CD33-targeted CAR-NK cell therapy for relapsed/refractory AML: preclinical evaluation and phase I trial. Exp. Hematol. Oncol. (2025) 14:1. doi: 10.1186/s40164-024-00592-6, PMID: 39748428 PMC11694373

[163] Wagner J Pfannenstiel V Waldmann A Bergs JWJ Brill B Huenecke S . A Two-Phase Expansion Protocol Combining Interleukin (IL)-15 and IL-21 Improves Natural Killer Cell Proliferation and Cytotoxicity against Rhabdomyosarcoma. Front. Immunol. (2017) 8:676. doi: 10.3389/fimmu.2017.00676, PMID: 28659917 PMC5466991

[164] Zhu H Blum RH Bernareggi D Ask EH Wu Z Hoel HJ . Metabolic reprogramming via deletion of CISH in human iPSC-derived NK cells promotes *in vivo* persistence and enhances anti-tumor activity. Blood. (2020) 135:399–410. doi: 10.1182/blood.2019000621, PMID: 32531207 PMC7415618

[165] Hood T Slingsby F Sandner V Geis W Schmidberger T Bevan N . A quality-by-design approach to improve process understanding and optimise the production and quality of CAR-T cells in automated stirred-tank bioreactors. Front. Immunol. (2024) 15:1335932. doi: 10.3389/fimmu.2024.1335932, PMID: 38655265 PMC11035805

[166] Zhang C Burger MC Jennewein L Genβler Schönfeld K Zeiner P . ErbB2/HER2-specific NK cells for targeted therapy of glioblastoma. J Natl Cancer Institute. (2016) 108. doi: 10.1093/jnci/djv375, PMID: 26640245

[167] Mallamaci R Budriesi R Clodoveo ML Biotti G Micucci M Ragusa A . Olive tree in circular economy as a source of secondary metabolites active for human and animal health beyond oxidative stress and inflammation. Molecules. (2021) 26:1072. doi: 10.3390/molecules26041027, PMID: 33670606 PMC7922482

[168] Huang S Xing F Dai Y Zhang Z Zhou G Yang S . Navigating chimeric antigen receptor-engineered natural killer cells as drug carriers via three-dimensional mapping of the tumor microenvironment. J Controlled Release. (2023) 362:524–35. doi: 10.1016/j.jconrel.2023.09.007, PMID: 37673307

[169] Kararoudi MN Likhite S Elmas E Yamamoto K Schwartz M Sorathia K . CRISPR-targeted CAR gene insertion using cas9/RNP and AAV6 enhances anti-AML activity of primary NK cells. Front Immunol. (2021) 12:763845. doi: 10.3389/fimmu.2021.763845

[170] Wang J Toregrosa-Allen S Elzey BD Utturkar S Lanman NA Bernal-Crespo V . Multispecific targeting of glioblastoma with tumor microenvironment-responsive multifunctional engineered NK cells. Proc Natl Acad Sci United States America. (2021) 118:e2107507118. doi: 10.1073/pnas.2107507118, PMID: 34740973 PMC8609337

[171] Han J Chu J Wang KC Zhang J Wang Y Colon JB . CAR-engineered NK cells targeting wild-type EGFR and EGFRvIII enhance killing of glioblastoma and patient-derived glioblastoma stem cells. Sci Rep. (2015) 5:11483. doi: 10.1038/srep11483, PMID: 26155832 PMC4496728

[172] Zuo P Li Y He C Wang T Zheng X Liu H . Anti-tumor efficacy of anti-GD2 CAR NK-92 cells in diffuse intrinsic pontine gliomas. Front Immunol. (2023) 14:1145706. doi: 10.3389/fimmu.2023.1145706, PMID: 37251413 PMC10213244

[173] Chang L Chitrakar C Nouri M . 3D Nanochannel Electroporation for Macromolecular Nucleotide Delivery. Methods Mol. Biol. (2020) 2050:69–77. doi: 10.1007/978-1-4939-9740-4_7, PMID: 31468480

[174] Dos Santos MH de Azevedo JTC da Silva Januário ME Schmidt DF Tirapelle MC Biggi AFB . 2B4 co-stimulation and dasatinib modulation enhance anti-CD19 CAR-NK-92 cell cytotoxicity. Front. Immunol. (2025) 16:1675877. doi: 10.3389/fimmu.2025.1675877, PMID: 41459521 PMC12741080

[175] Lehmann C Zeis M Uharek L . Activation of natural killer cells with interleukin 2 (IL-2) and IL-12 increases perforin binding and subsequent lysis of tumour cells. Br J Haematology. (2001) 114:660–5. doi: 10.1046/j.1365-2141.2001.02995.x, PMID: 11552995

[176] Huntington ND Legrand N Alves NL Jaron B Weijer K Plet A . IL-15 trans-presentation promotes human NK cell development and differentiation *in vivo*. J Exp Med. (2009) 206:25–34. doi: 10.1084/jem, PMID: 19103877 PMC2626663

[177] Xu X Cao P Wang M Wan Y Sun S Chen Y . Signaling intact membrane-bound IL-15 enables potent anti-tumor activity and safety of CAR-NK cells. Front. Immunol. (2025) 16:1658580. doi: 10.3389/fimmu.2025.1658580, PMID: 41098734 PMC12518233

[178] Pinette A McMichael E Courtney NB Duggan M Benner BN Choueiry F . An IL-15-based superagonist ALT-803 enhances the NK cell response to cetuximab-treated squamous cell carcinoma of the head and neck. Cancer Immunol. Immunother. (2019) 68:1379–89. doi: 10.1007/s00262-019-02372-2, PMID: 31338557 PMC7032639

[179] Mathios D Park C-K Marcus WD Alter S Rhode P Jeng E . Therapeutic administration of IL-15 superagonist complex ALT-803 leads to long-term survival and durable antitumor immune response in a murine glioblastoma model. Int J Cancer. (2016) 138:187–94. doi: 10.1002/ijc.29686, PMID: 26174883 PMC4696021

[180] Vallera DA Felices M McElmurry R McCullar V Zhou X Schmohl J . IL15 trispecific killer engagers (TriKE) make natural killer cells specific to CD33+ Targets while also inducing persistence, vivo expansion, and enhanced function. Clin Cancer Res. (2016) 22:3440–50. doi: 10.1158/1078-0432.CCR-15-2710, PMID: 26847056 PMC4947440

[181] Miller JS Felice M McElmurry R McCullar V Zhou X Jakub Tolar J . Trispecific Killer Engagers (TriKEs) that contain IL-15 to make NK cells antigen specific and to sustain their persistence and expansion. Blood. (2015) 126:232. doi: 10.1182/blood.V126.23.232.232

[182] Elmas E Saljoughian N Pereira M Tullius BP Sorathia K Nakkula RJ . CRISPR Gene Editing of Human Primary NK and T Cells for Cancer Immunotherapy. Front. Oncol. (2022) 12:834002. doi: 10.3389/fonc.2022.834002, PMID: 35449580 PMC9016158

[183] Yang Y Yang F Huang Z Li Y Shi H Sun Q . T cells, NK cells, and tumor-associated macrophages in cancer immunotherapy and the current state of the art of drug delivery systems. Front Immunol. (2023) 14:1199173. doi: 10.3389/fimmu.2023.1199173, PMID: 37457707 PMC10348220

[184] Cichocki F Valamehr B Bjordahl R Zhang B Rezner B Rogers P . GSK3 inhibition drives maturation of NK cells and enhances their antitumor activity. Cancer Res. (2017) 77:5664–75. doi: 10.1158/0008-5472.CAN-17-0799, PMID: 28790065 PMC5645243

[185] Cifaldi L Locatelli F Marasco E Moretta L Pistoia V . Boosting natural killer cell-based immunotherapy with anticancer drugs: a perspective. Trends Mol Med. (2017) 23:1156–75. doi: 10.1016/j.molmed.2017.10.002, PMID: 29133133

[186] Guerrero JA Klysz DD Chen Y Malipatlolla M Lone J Fowler C . GLUT1 overexpression in CAR-T cells induces metabolic reprogramming and enhances potency. Nat. Commun. (2024) 15:8658. doi: 10.1038/s41467-024-52666-y, PMID: 39370422 PMC11456602

[187] Du M Yu T Zhan Q Li H Zou Y Geng M . Development of a novel lactate dehydrogenase A inhibitor with potent antitumor activity and immune activation. Cancer Sci. (2022) 113:2974–85. doi: 10.1111/cas.15468, PMID: 35722994 PMC9459323

[188] Harmon C Robinson MW Hand F . Lactate-mediated acidification of tumor microenvironment induces apoptosis of liver-resident NK cells in colorectal liver metastasis. Cancer Immunol Res. (2019) 7:335–46. doi: 10.1158/2326-6066.CIR-18-0481, PMID: 30563827

[189] Zhi L Zhang Z Gao Q Shang C He W Wang Y . CAR-NK cells with dual targeting of PD-L1 and MICA/B in lung cancer tumor models. BMC Cancer. (2025) 25:337. doi: 10.1186/s12885-025-13780-2., PMID: 40000974 PMC11853679

[190] Hamieh M Dobrin A Cabriolu A Stegen S Giavridis T Mansilla-Soto J . CAR T cell trogocytosis and cooperative killing regulate tumour antigen escape. Nature. (2019) 568:112–6. doi: 10.1038/s41586-019-1054-1, PMID: 30918399 PMC6707377

[191] Li Y Basar R Wang G Liu E Moyes JS Li L . KIR-based inhibitory CARs overcome CAR-NK cell trogocytosis-mediated fratricide and tumor escape. Nat Med. (2022) 28:2133–44. doi: 10.1038/s41591-022-02003-x, PMID: 36175679 PMC9942695

[192] Alishah K Birtel M Masoumi E Jafarzadeh L Mirzaee HR Hadjati J . CRISPR/Cas9-mediated TGFβRII disruption enhances anti-tumor efficacy of human chimeric antigen receptor T cells in vitro. J. Transl. Med. (2021) 19:482. doi: 10.1186/s12967-021-03146-0, PMID: 34838059 PMC8627098

[193] Ng YY Tay JCK Wang S . CXCR1 expression improves anti-cancer efficacy of intravenously injected CAR−NK cells in mice with peritoneal xenografts. Mol Ther - Oncolytics. (2020) 19:234–44. doi: 10.1016/j.omto.2020.08.005, PMID: 31970285 PMC6965500

[194] Zhao L Zhang Y Yang X Chen T Liu J Guo Z . Nattokinase-driven remodeling of tumor microenvironment enhances the efficacy of MSLN-targeted CAR-T cell therapy in solid tumors. Cancer Immunol. Immunother. (2025) 74:315. doi: 10.1007/s00262-025-04167-0, PMID: 41021005 PMC12480323

[195] Cichocki F Goodridge JP Bjordahl R Mahmood S Davis ZB Gaidarova S . Dual antigen-targeted off-the-shelf NK cells show durable response and prevent antigen escape in lymphoma and leukemia. Blood. (2022) 140:2451–62. doi: 10.1182/blood.2021015184, PMID: 35917442 PMC9918847

[196] Klapdor R Wang S Morgan M Dörk T Hacker U Hillemanns R . Characterization of a novel third-generation anti-CD24-CAR against ovarian cancer. Int J Mol Sci. (2019) 20:660. doi: 10.3390/ijms20030660, PMID: 30717444 PMC6387114

[197] Gonzalez VD Huang Y-W Delgado-Gonzalez A Chen S-Y Donoso K Sachs K . High-grade serous ovarian tumor cells modulate NK cell function to create an immune-tolerant microenvironment. Cell Rep. (2021) 36:109632. doi: 10.1016/j.celrep.2021.109632, PMID: 34469729 PMC8546503

[198] Kim J Park S Kim J Kim Y Yoon HM Rayhan BR . Trogocytosis-mediated immune evasion in the tumor microenvironment. Exp. Mol. Med. (2025) 57:1–12. doi: 10.1038/s12276-024-01364-2, PMID: 39741180 PMC11799389

